# Comorbidity Burden and Cumulative Anti-HER2 Exposure Associated with Cardiac Dysfunction in Breast Cancer: A Real-World Cardio-Oncology Cohort

**DOI:** 10.3390/jcm15197700

**Published:** 2026-10-05

**Authors:** Andrea Sonaglioni, Emanuela Fossile, Maria Adelaide Pessi, Maria Gemelli, Massimo Baravelli, Michele Lombardo, Gian Luigi Nicolosi, Barbara Bassani, Antonino Bruno, Paola Muti

**Affiliations:** 1 Division of Cardiology, IRCCS MultiMedica, 20123 Milan, Italy; massimo.baravelli@multimedica.it (M.B.); michele.lombardo@multimedica.it (M.L.); 2 Division of Oncology, IRCCS MultiMedica, 20123 Milan, Italy; emanuela.fossile@multimedica.it (E.F.); mariaadelaide.pessi@multimedica.it (M.A.P.); maria.gemelli@multimedica.it (M.G.); 3 Division of Cardiology, Policlinico San Giorgio, 33170 Pordenone, Italy; gianluigi.nicolosi@gmail.com; 4 Laboratory of Innate Immunity, IRCCS MultiMedica, 20138 Milan, Italy; barbara.bassani@multimedica.it (B.B.); antonino.bruno@uninsubria.it (A.B.); 5 Laboratory of Translational Immunology, Centro di Ricerca per L’invecchiamento di Successo (CRIS), Department of Biotechnology and Life Sciences, University of Insubria, 21100 Varese, Italy; 6 Department of Biomedical, Surgical and Dental Sciences, University of Milan, 20122 Milan, Italy; paola.muti@unimi.it; 7 IRCCS MultiMedica, 20138 Milan, Italy

**Keywords:** cardio-oncology, cardiotoxicity, cancer-therapy-related cardiac dysfunction, HER2, trastuzumab, pertuzumab, Charlson Comorbidity Index, global longitudinal strain, left ventricular ejection fraction

## Abstract

**Background:** Cancer-therapy-related cardiac dysfunction (CTRCD) remains an important complication of human epidermal growth factor receptor 2 (HER2)-targeted therapy in patients with breast cancer. Although conventional cardiovascular risk factors and serial cardiac imaging are routinely incorporated into cardio-oncology surveillance, the potential contribution of global comorbidity burden and cumulative anti-HER2 treatment exposure remains incompletely characterized. We investigated the occurrence, clinical course, and factors associated with CTRCD in a large real-world population of patients with breast cancer receiving HER2-targeted therapy. **Methods:** Adult patients with breast cancer exposed to HER2-targeted therapy and undergoing longitudinal cardio-oncology surveillance at a single tertiary center were retrospectively identified over a 10-year accrual period (January 2014–December 2024). Mean individual follow-up was approximately 2.0 years (range, 0.5–9.0 years). CTRCD was defined according to the harmonized International Cardio-Oncology Society (IC-OS) definitions incorporated into the 2022 ESC Guidelines on cardio-oncology, integrating left ventricular ejection fraction (LVEF), left ventricular global longitudinal strain (LV-GLS), and cardiac biomarkers when available. Baseline comorbidity burden was quantified using the Charlson Comorbidity Index (CCI), while cumulative anti-HER2 exposure was quantified according to the total number of treatment cycles. Factors associated with CTRCD were investigated using univariable and parsimonious multivariable logistic regression. **Results:** A total of 850 patients with breast cancer receiving anti-HER2 therapy were included. CTRCD occurred in 179 patients (21.1%) and was predominantly mild, whereas clinically overt heart failure occurred in 2.9%. LVEF and LV-GLS deteriorated at nadir and subsequently showed substantial recovery during follow-up. Conventional cardiovascular risk factors, previous anthracycline exposure, tumor grade, baseline LVEF, and baseline LV-GLS were not significantly associated with CTRCD. In multivariable analysis, increasing CCI (adjusted OR 1.35 per one-point increase, 95% CI 1.12–1.63; *p* = 0.002) and cumulative anti-HER2 exposure (adjusted OR 1.41 per five additional cycles, 95% CI 1.10–1.80; *p* = 0.006) remained associated with CTRCD after mutual adjustment. Progressively higher observed proportions of CTRCD were found across increasing CCI categories and cumulative anti-HER2 exposure levels. **Conclusions:** In this large real-world cohort assembled over a 10-year accrual period, CTRCD was relatively frequent but predominantly mild and reversible. Greater global comorbidity burden and cumulative anti-HER2 exposure were associated with a higher occurrence of CTRCD. However, the association with cumulative anti-HER2 exposure should be considered hypothesis-generating rather than causal because of potential time-dependent and reverse-causality bias. Prospective validation is required to determine their potential role in individualized cardiovascular surveillance.

## 1. Introduction

Advances in cancer diagnosis and treatment have substantially improved survival across a broad spectrum of malignancies, resulting in a growing population of patients exposed to potentially cardiotoxic therapies [1,2,3]. Consequently, cardiovascular complications have emerged as an important determinant of morbidity, treatment discontinuation, and long-term prognosis among cancer patients and survivors [4,5,6]. Cancer-therapy-related cardiac dysfunction (CTRCD) represents one of the most clinically relevant manifestations of cardiovascular toxicity, encompassing a continuum from asymptomatic myocardial dysfunction to overt heart failure [7,8,9]. Contemporary cardio-oncology has therefore progressively shifted from the treatment of established cardiac injury toward its early identification and prevention through baseline cardiovascular risk assessment, longitudinal imaging surveillance, cardiac biomarkers, and timely implementation of cardioprotective strategies [10,11,12].

Human epidermal growth factor receptor 2 (HER2)-targeted therapies represent a paradigmatic yet challenging setting in which this preventive approach is particularly relevant. Trastuzumab and other HER2-directed agents have profoundly improved oncological outcomes in patients with HER2-positive breast cancer in the neoadjuvant, adjuvant, and metastatic settings [13,14,15]. However, interference with HER2-dependent myocardial signaling may impair cardiomyocyte adaptive and repair mechanisms, exposing susceptible patients to left ventricular dysfunction during treatment [16,17,18]. Although anti-HER2-related cardiotoxicity is frequently reversible when promptly recognized and appropriately managed, its occurrence may require treatment interruption or modification and may compromise the optimal delivery of anticancer therapy [19,20,21]. Accordingly, serial assessment of left ventricular ejection fraction (LVEF), left ventricular global longitudinal strain (LV-GLS), and, when appropriate, cardiac biomarkers has become an integral component of cardiovascular surveillance during HER2-targeted treatment [22,23,24,25].

Nevertheless, prediction of CTRCD remains challenging. Cardiotoxicity is unlikely to result from a single determinant and may instead reflect the interaction between baseline patient characteristics, pre-existing cardiovascular disease, conventional cardiovascular risk factors, concomitant comorbidities, previous cardiotoxic treatments, and the intensity and duration of ongoing anticancer therapy [26,27,28]. In routine cardio-oncology practice, these factors frequently coexist, particularly in older and medically complex patients. Consequently, assessment of individual cardiovascular risk factors alone may provide an incomplete representation of the overall vulnerability of a patient undergoing potentially cardiotoxic treatment. A more comprehensive evaluation of comorbidity burden may therefore complement conventional cardiovascular risk stratification.

The Charlson Comorbidity Index (CCI) is a simple and widely applicable instrument that summarizes the burden of multiple clinically relevant comorbidities within a single score [29]. Although originally developed to estimate mortality according to comorbidity burden [30], its potential utility may extend to clinical settings in which reduced physiological reserve could influence susceptibility to treatment-related complications [31]. In cardio-oncology, such an integrated measure may be particularly attractive because patients often present with heterogeneous combinations of cardiovascular and non-cardiovascular conditions [32,33]. However, the potential relationship between increasing global comorbidity burden and the development of CTRCD during contemporary anticancer treatment remains insufficiently characterized [34].

Treatment-related exposure represents another potentially important component of cardiotoxic risk. Previous anthracycline exposure is an established determinant of cardiovascular toxicity [35,36,37], whereas risk stratification during HER2-targeted therapy has traditionally relied largely on baseline cardiovascular characteristics and serial changes in cardiac function during treatment [38,39]. However, among patients receiving HER2-targeted agents, cumulative exposure may vary considerably according to disease stage, treatment setting, therapeutic response, recurrence, and metastatic progression [40,41,42]. Consequently, the cardiovascular impact of HER2-targeted therapy may not be adequately captured by considering treatment exposure as a simple dichotomous variable. Whether the cumulative duration of anti-HER2 therapy, expressed as the number of administered treatment cycles, provides additional information for identifying patients at increased risk of CTRCD remains insufficiently characterized [43]. Clarifying this relationship may contribute to a more dynamic assessment of cardiotoxic risk and support individualized cardiovascular surveillance throughout the oncological treatment pathway.

Therefore, the present study specifically focused on a large cohort of patients with breast cancer exposed to HER2-targeted therapy and was designed to comprehensively characterize the incidence, severity, echocardiographic features, clinical management, and recovery of CTRCD over a 10-year observation period. We also sought to investigate clinical, oncological, treatment-related, and baseline echocardiographic factors associated with CTRCD within this anti-HER2-treated breast cancer population. Particular attention was devoted to the potential contribution of overall comorbidity burden, assessed by the CCI, and cumulative anti-HER2 treatment exposure. By integrating baseline patient characteristics with the longitudinal burden of HER2-targeted treatment, the study aimed to identify readily accessible factors associated with CTRCD that may provide additional information for longitudinal cardiovascular risk assessment and help inform surveillance strategies in patients with breast cancer receiving anti-HER2 therapy.

## 2. Materials and Methods

### 2.1. Study Design and Population

This study represents a focused analysis of the retrospective component of the CARTOX-PREDICT project, an observational, retro-prospective, single-center cardio-oncology study conducted at IRCCS MultiMedica, Ospedale San Giuseppe, Milan, Italy. The original CARTOX-PREDICT study protocol, provided in Supplementary Material File S1 [25,26], was designed to investigate cardiovascular toxicity associated with different contemporary anticancer treatments, including anthracyclines, HER2-targeted therapies, fluoropyrimidines, vascular endothelial growth factor inhibitors, and immune checkpoint inhibitors.

For the present investigation, we specifically selected the subpopulation of adult patients with breast cancer exposed to HER2-targeted therapy and undergoing longitudinal cardio-oncology surveillance. Thus, unlike the broader CARTOX-PREDICT population, the current study was restricted to patients with breast cancer who had received anti-HER2 treatment and had serial cardiac assessments available. Patients were identified from the institutional oncology and cardiology databases over the 10-year study period, from 1 January 2014 to 31 December 2024.

Eligible patients were aged > 18 years, had a diagnosis of breast cancer, and had received at least one HER2-targeted agent, predominantly trastuzumab alone or in combination with pertuzumab, in the neoadjuvant, adjuvant, maintenance, and/or metastatic setting. HER2-targeted treatment could be administered alone or in combination or sequence with other systemic anticancer therapies. To permit longitudinal assessment of cardiac function, patients were required to have a baseline cardiovascular evaluation performed before or at the initiation of HER2-targeted therapy and at least one subsequent echocardiographic examination during follow-up. Patients for whom only a baseline echocardiographic examination was available, without any subsequent cardiac imaging assessment, as well as those with insufficient clinical documentation to reliably establish treatment exposure or cardiovascular outcomes, were excluded.

### 2.2. Data Collection and Clinical Assessment

Demographic, anthropometric, cardiovascular, oncological, laboratory, therapeutic, and echocardiographic information was retrospectively retrieved from institutional electronic medical records and cardio-oncology archives. The original protocol established collection of baseline information before anticancer treatment together with serial follow-up data obtained during treatment.

Recorded clinical variables included age at initiation of cancer therapy, sex, body mass index (BMI), conventional cardiovascular risk factors—including hypertension, diabetes mellitus, dyslipidemia, and current or previous smoking—and pre-existing cardiovascular disease. Relevant non-cardiovascular comorbidities were also systematically reviewed.

Rather than considering comorbid conditions exclusively as isolated variables, overall comorbidity burden was summarized using the CCI [30]. The CCI was calculated from comorbid conditions documented at baseline and was subsequently analyzed both as a continuous variable and according to clinically interpretable categories (CCI = 2, CCI 3–4, and CCI ≥ 5).

Laboratory variables included complete blood cell count, renal and hepatic function, electrolytes, C-reactive protein, conventional cardiac troponin I (cTnI; non-high-sensitivity assay; institutional upper reference limit, 0.04 ng/mL), and N-terminal pro-B-type natriuretic peptide (NT-proBNP), when available.

### 2.3. Oncological Characteristics and Treatment Exposure

Tumor-related information included primary cancer site, histological subtype, tumor grade, hormone-receptor expression, HER2 status, Ki-67 proliferation index, disease stage, and oncological status during follow-up, whenever available. Data regarding systemic anticancer treatment, radiotherapy, and endocrine therapy were systematically retrieved from the institutional medical records.

Detailed treatment history was reconstructed for each patient, including previous or concomitant exposure to anthracyclines and taxanes and the use of HER2-targeted agents. For HER2-directed therapy, exposure to trastuzumab and pertuzumab was recorded together with the treatment setting and duration. HER2-targeted therapy could be administered in the neoadjuvant or adjuvant setting or, in patients with advanced or metastatic disease, continued according to oncological indication until disease progression, unacceptable toxicity, or treatment discontinuation.

To provide a quantitative measure of treatment burden, cumulative anti-HER2 exposure was determined from the total number of treatment cycles received during the observation period. All documented anti-HER2 cycles administered across the neoadjuvant, adjuvant, metastatic, and maintenance settings were included in the cumulative exposure variable. Exposure within individual treatment settings was recorded separately when available, and the overall cumulative number of anti-HER2 cycles across all treatment phases was subsequently derived for each patient. Temporary or permanent interruption of HER2-targeted treatment because of suspected or established cardiotoxicity was also documented. Information regarding subsequent resumption of anticancer therapy, treatment completion, disease progression, and oncological outcome was collected throughout follow-up.

### 2.4. Transthoracic Echocardiography

Transthoracic echocardiography (TTE) was performed as part of routine cardio-oncology surveillance. All echocardiographic examinations were performed using the same ultrasound system (Sparq, Philips Healthcare, Andover, MA, USA) throughout the study period. Examinations were acquired with patients in the left lateral decubitus position, and measurements were obtained in accordance with contemporary recommendations for cardiac chamber quantification and assessment of LV diastolic function [44,45]. The majority of echocardiographic examinations were performed and interpreted by the same experienced cardiologist (A.S.), whereas a smaller proportion was performed by a second experienced operator (M.B.), thereby ensuring substantial consistency in image acquisition and measurement methodology throughout the study period.

The present analysis focused on echocardiographic variables that could be consistently retrieved across the retrospective study period. These included interventricular septal thickness, LV end-diastolic diameter, left atrial volume, transmitral E/A ratio, E/e’ ratio, LV ejection fraction (LVEF), and pulmonary artery systolic pressure (PASP). LVEF was assessed using the biplane modified Simpson method whenever technically feasible.

For longitudinal assessment, LVEF was recorded at three clinically relevant time points: baseline, defined as the examination performed before or at initiation of anticancer treatment; nadir, defined as the lowest documented LVEF during oncological treatment and cardio-oncology follow-up; and final follow-up, corresponding to the last available echocardiographic examination. The absolute change in LVEF from baseline to nadir was calculated for each patient. In accordance with the 2022 ESC Guidelines on cardio-oncology [25], a decline in LVEF of ≥10 percentage points from baseline and the occurrence of an LVEF <50% at nadir were additionally recorded as categorical variables for the subsequent assessment of LV systolic dysfunction.

### 2.5. Assessment of Myocardial Deformation

Speckle-tracking echocardiography (STE) was used for the assessment of LV myocardial deformation. Two-dimensional grayscale images acquired from the standard apical four-chamber, two-chamber, and three-chamber views were used for longitudinal strain analysis, in accordance with the methodology prespecified in the CARTOX-PREDICT protocol. All strain analyses included in the present study were performed offline by the same experienced operator (A.S.) using Philips QLAB software, version 10.3.1 (Philips Healthcare, Andover, MA, USA), thereby avoiding inter-observer variability related to post-processing.

For each apical view, the LV endocardial border was identified and the region of interest was adjusted when necessary to ensure adequate tracking of the myocardial wall throughout the cardiac cycle. Segmental longitudinal strain values obtained from the three apical views were combined to generate the LV-GLS, together with the corresponding bull’s-eye representation. This approach was consistent with standardized recommendations for two-dimensional STE [46,47].

For longitudinal assessment, LV-GLS was evaluated at baseline, at the examination corresponding to the lowest documented myocardial deformation during treatment (GLS nadir), and at the last available follow-up examination. The relative change in GLS from baseline to nadir was calculated for each patient. A new relative decline in LV-GLS >15% from baseline was considered clinically relevant and was incorporated into the assessment of CTRCD in accordance with contemporary European cardio-oncology criteria [25].

Because of the retrospective nature and extended duration of the study, adequate images for longitudinal strain analysis were not uniformly available at all time points in every patient. Therefore, GLS was analyzed whenever technically feasible and was interpreted in conjunction with serial LVEF measurements and the overall clinical assessment.

### 2.6. Definition and Grading of Cancer-Therapy-Related Cardiac Dysfunction

CTRCD was defined and graded according to the harmonized International Cardio-Oncology Society (IC-OS) definitions endorsed and incorporated into the 2022 ESC Guidelines on cardio-oncology [25], integrating changes in LVEF, LV-GLS, and cardiac biomarkers when available.

Mild asymptomatic CTRCD was defined by preserved LVEF (≥50%) accompanied by a new relative reduction in LV-GLS >15% from baseline and/or a new elevation in cardiac troponin I (cTnI) above the institutional upper reference limit. Moderate asymptomatic CTRCD was defined by a new LVEF reduction ≥10 percentage points resulting in an LVEF of 40–49%, or by an LVEF reduction <10 percentage points resulting in an LVEF of 40–49% when accompanied by a >15% relative LV-GLS decline and/or a new elevation in cTnI above the institutional upper reference limit. Severe asymptomatic CTRCD was defined as a new reduction in LVEF to <40%. Symptomatic CTRCD was identified when cardiac dysfunction was accompanied by clinical manifestations of heart failure.

For cardiac biomarker assessment, a new elevation above the institutional upper reference limit was considered abnormal. Conventional cTnI (non-high-sensitivity assay) was used, with an institutional upper reference limit of 0.04 ng/mL. Cardiac biomarker information was incorporated into CTRCD classification when available; however, biomarkers were not systematically measured at all time points because of the retrospective nature and extended duration of the study.

When LV-GLS or cardiac biomarker measurements were unavailable, missing values were not considered normal; rather, the unavailable component was not used for CTRCD classification, which was based on the remaining available criteria. Consequently, moderate or severe CTRCD could still be identified according to LVEF-based criteria, whereas ascertainment of mild asymptomatic CTRCD was more dependent on the availability of LV-GLS and/or cardiac biomarker data.

For each patient developing CTRCD, the severity, echocardiographic pattern, clinical heart-failure status, modification or interruption of anticancer treatment, initiation of cardioprotective therapy, and subsequent recovery of LV systolic function were recorded.

### 2.7. Management of CTRCD and LVEF Recovery

Clinical management following CTRCD was reconstructed from cardiology and oncology records. Recorded interventions included temporary or permanent interruption of anticancer treatment and initiation or intensification of cardiovascular therapy, including angiotensin-converting enzyme inhibitors, angiotensin receptor blockers or angiotensin receptor–neprilysin inhibitors, beta-blockers, mineralocorticoid receptor antagonists, sodium–glucose cotransporter-2 (SGLT2) inhibitors, and diuretics when clinically indicated.

LV systolic recovery was classified as complete, partial, or absent according to the subsequent echocardiographic course, based on the previously established thresholds for clinically significant changes in cardiac function (≥10 percentage points for LVEF and >15% for LV-GLS). When temporal information was sufficiently reliable, the interval between CTRCD/LVEF nadir and the first examination documenting recovery was also recorded.

### 2.8. Study Outcomes

The primary outcome of the present study was the occurrence of CTRCD during longitudinal follow-up in patients with breast cancer receiving HER2-targeted therapy, according to the predefined criteria described above.

Secondary outcomes included CTRCD severity; occurrence of clinically overt heart failure; magnitude of LVEF and LV-GLS deterioration during treatment; subsequent recovery of → LV systolic function; temporary or permanent interruption of HER2-targeted therapy because of cardiotoxicity; initiation or intensification of cardioprotective treatment; and time to recovery of cardiac function when available.

An additional exploratory objective was to identify clinical, oncological, treatment-related, and baseline echocardiographic factors associated with CTRCD. Particular attention was devoted to the potential contribution of baseline comorbidity burden, quantified by the CCI, and cumulative anti-HER2 exposure, quantified → according to the total number of treatment cycles received. These analyses were intended to explore whether patient-related vulnerability and treatment-related exposure were associated with CTRCD and could provide additional information for longitudinal cardiovascular risk assessment within the anti-HER2-treated breast cancer population.

### 2.9. Statistical Analysis

Initial data screening was performed to identify inconsistencies, implausible values, and missing observations before statistical analysis. The distribution of continuous variables was assessed using the Kolmogorov–Smirnov test together with visual inspection of Q–Q plots. Normally distributed continuous variables are presented as mean ± standard deviation (SD), whereas non-normally distributed variables are reported as median and interquartile range [IQR]. Categorical variables are expressed as absolute numbers and percentages. Analyses involving variables with missing observations were performed using available cases, without imputation of missing values unless otherwise specified.

The occurrence of CTRCD during follow-up was considered the dependent variable for regression analyses. Candidate variables were selected among baseline demographic and cardiovascular characteristics, global comorbidity burden, oncological variables, treatment-related exposures, and baseline echocardiographic parameters. Associations between individual candidate variables and CTRCD were initially examined using univariable binary logistic regression. For each predictor, odds ratios (ORs), corresponding 95% confidence intervals (CIs), and *p* values were calculated. Continuous variables were modeled using clinically interpretable increments whenever appropriate. Age was evaluated per 10-year increment, body mass index per 5 kg/m^2^ increment, and the CCI per one-point increment. Cumulative anti-HER2 exposure was quantified as the total number of treatment cycles administered during the oncological treatment course and modeled per five additional cycles. Baseline LVEF and LV-GLS were modeled per one-percentage-point decrease. For ease of interpretation, LV-GLS values were expressed as positive absolute values throughout the statistical analyses, with lower values indicating worse myocardial deformation. The use of univariable and multivariable logistic regression to investigate factors associated with CTRCD was prespecified in the original CARTOX-PREDICT study protocol (Supplementary Material File S1), and the present analytical approach was therefore consistent with the predefined statistical plan. A time-to-event analysis using Cox proportional hazards regression was not performed because the retrospective dataset did not permit sufficiently reliable ascertainment of the exact date of first CTRCD occurrence for all affected patients or reconstruction of anti-HER2 exposure with adequate temporal granularity. In routine clinical practice, cardiac assessments were performed intermittently, commonly at intervals of approximately 1–3 months, such that CTRCD could generally be localized to an interval between consecutive assessments rather than to a precise date of onset. Consequently, the temporal relationship between cumulative anti-HER2 exposure and CTRCD could not be modeled with sufficient accuracy to support a Cox analysis incorporating treatment exposure as a time-dependent covariate.

To identify factors associated with CTRCD after mutual adjustment, variables showing the strongest associations at univariable analysis and considered clinically relevant were subsequently evaluated in a parsimonious multivariable binary logistic regression model. The final model was deliberately restricted to CCI and cumulative anti-HER2 exposure to address the exploratory objective of assessing their mutually adjusted associations with CTRCD, rather than to develop a comprehensive prediction model. With 179 CTRCD events and two covariates, the resulting events-per-variable ratio was 89.5; therefore, model restriction was not driven by an events-per-variable constraint. Adjusted odds ratios (aORs) with corresponding 95% CIs were calculated.

Two complementary secondary analyses were performed to further characterize the relationships observed for the variables retained in the multivariable model. First, patients were stratified according to baseline comorbidity burden into three predefined CCI categories: CCI = 2, CCI 3–4, and CCI ≥ 5. CTRCD occurrence was evaluated across progressively higher CCI categories to assess the presence of a graded relationship between comorbidity burden and cardiotoxicity. Second, cumulative anti-HER2 exposure was categorized as <10 cycles, 10–16 cycles, and ≥17 cycles, allowing the relationship between increasing treatment exposure and CTRCD to be examined across clinically interpretable treatment-duration strata. These categorical analyses complemented the continuous-variable logistic regression models and were primarily intended to facilitate clinical interpretation of the observed risk gradients.

All statistical analyses were performed using IBM SPSS Statistics for Windows, version 28.0 (IBM Corp., Armonk, NY, USA). All statistical tests were two-sided, and a *p* value < 0.05 was considered statistically significant.

### 2.10. Ethical Considerations

The CARTOX-PREDICT study was conducted in accordance with the principles of the Declaration of Helsinki and applicable standards for clinical research. The study protocol was approved by the Comitato Etico Territoriale Lombardia 5 (CET Lombardia 5; Protocol No. 678/25), with final ethical clearance granted on 9 May 2026. The official Ethics Committee approval document is provided in the Supplementary Materials, File S2. Patient information was handled in anonymized form and in accordance with institutional requirements for confidentiality, privacy, and data protection.

## 3. Results

### 3.1. Study Population and Baseline Clinical Characteristics

A total of 850 adult patients with breast cancer exposed to HER2-targeted therapy and undergoing longitudinal cardio-oncology surveillance at IRCCS MultiMedica, Ospedale San Giuseppe, Milan, Italy, between 1 January 2014 and 31 December 2024 were included in the present analysis. The mean duration of follow-up was approximately 2.0 years (range, 0.5–9.0 years).

The study population was almost entirely composed of women and had a mean age in the mid-fifties at initiation of cancer therapy. The overall anthropometric profile was characterized by a normal mean BMI, although overweight and obese individuals were also represented. Hypertension was the most frequent conventional cardiovascular risk factor, followed by dyslipidemia and current or previous smoking, whereas diabetes mellitus was less common (Table 1).

As shown in Table 2, approximately one-third of patients had evidence of pre-existing cardiac disease.

Mitral valve prolapse (MVP)/billowing with or without mitral regurgitation represented the most frequently documented cardiac phenotype, followed by LV hypertrophy/hypertensive heart disease, aortic valve or aortic disease, arrhythmias or conduction disorders, and ischemic cardiovascular disease. The non-cardiovascular comorbidity profile was heterogeneous, with osteopenia/osteoporosis, thyroid disease, hepatic steatosis and metastatic cancer among the most frequently observed conditions. Overall comorbidity burden was relatively low in most patients, with a median CCI of 2; nevertheless, a clinically relevant subgroup showed intermediate or high CCI values.

Baseline laboratory assessment showed an overall preserved hematological, renal, hepatic, and electrolyte profile, without major abnormalities (Table 3). Because of the retrospective nature and extended study period, inflammatory and cardiac biomarkers were not systematically assessed, with CRP, conventional cTnI, and NT-proBNP available in approximately 70%, 60%, and 55% of patients, respectively.

### 3.2. Oncological Characteristics and Anticancer Treatments

The study population was entirely composed of patients with breast cancer, with a relatively balanced distribution between left- and right-sided tumors. Invasive ductal/no special type (NST) carcinoma represented the predominant histological subtype, and high-grade tumors were frequently observed. Approximately two-thirds of tumors expressed estrogen receptors, while the HER2 profile reflected the heterogeneous biological characteristics documented over the oncological disease course, including changes in HER2 expression or amplification at recurrence or metastatic progression (Table 4).

By study design, all patients included in the present cohort had been exposed to HER2-targeted therapy, predominantly trastuzumab-based treatment, while pertuzumab was administered in approximately one-fifth of patients. Exposure to antibody–drug conjugates, including trastuzumab emtansine (T-DM1) and trastuzumab deruxtecan (T-DXd), was less frequent. Anti-HER2 therapy was delivered across different phases of the oncological pathway, including neoadjuvant and adjuvant treatment, with a longer cumulative exposure generally observed in the adjuvant setting. The therapeutic background was highly multimodal: anthracyclines and taxanes were commonly administered, and a substantial proportion of patients also received radiotherapy and endocrine therapy. Overall, these treatment patterns reflect the complex sequential and combined therapeutic strategies encountered in contemporary management of HER2-targeted breast cancer (Table 5).

### 3.3. Echocardiographic Changes and Occurrence of CTRCD

Baseline echocardiography showed, on average, preserved LV chamber dimensions and systolic function. During longitudinal surveillance, a measurable reduction in LVEF was observed, with subsequent improvement toward baseline values at final follow-up. A similar temporal pattern was evident for myocardial deformation, with LV-GLS worsening at nadir and subsequently improving. Reliable longitudinal LV-GLS assessment was not feasible in approximately 100 patients (11.8% of the overall cohort), primarily because of inadequate acoustic windows and/or insufficient image quality for retrospective speckle-tracking analysis. Accordingly, GLS-derived longitudinal measurements and categorical changes should be interpreted as referring to patients with technically adequate images available for analysis. In contrast, indices of LV filling pressure and PASP remained comparatively stable during follow-up (Table 6).

**Figure 1:**
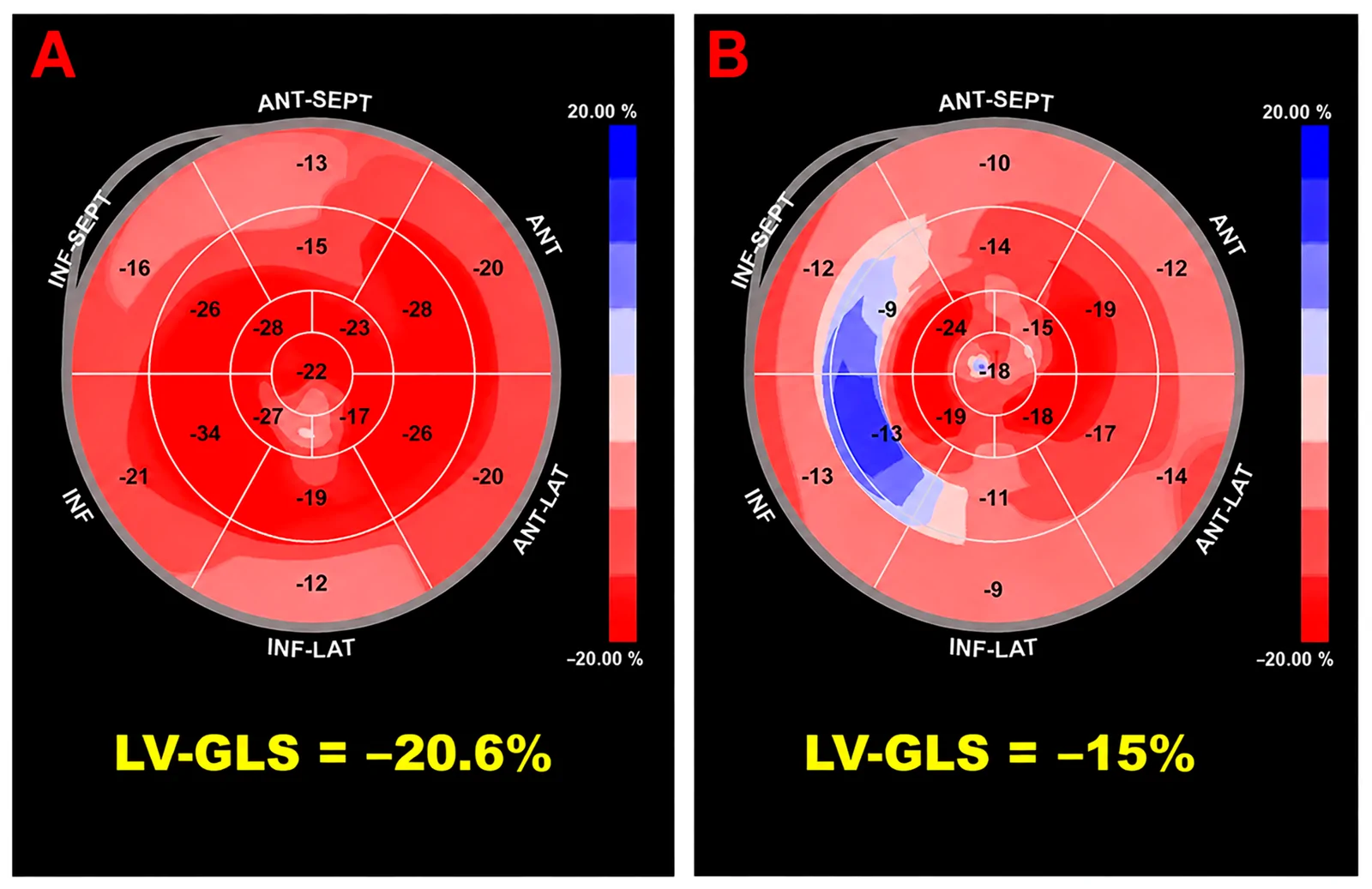
Representative LV speckle-tracking bull’s-eye plots illustrating anti-HER2-related left ventricular dysfunction before and during HER2-targeted therapy. (**A**) Baseline examination in a 51-year-old woman, showing preserved global longitudinal strain (LV-GLS, –20.6%) with relatively homogeneous myocardial deformation. (**B**) Follow-up examination performed 9 months after baseline, showing a moderate reduction in LV-GLS (–15%) with a predominantly regional pattern of dysfunction, characterized by more pronounced impairment of the inferior and inferoseptal territories, particularly at the mid-basal level, as reflected by the shift toward lighter blue color coding on the polar map. This example illustrates the regional pattern of myocardial involvement frequently observed among patients developing cancer-therapy-related cardiac dysfunction in the present cohort. HER2, human epidermal growth factor receptor 2; LV, left ventricular; LV-GLS, left ventricular global longitudinal strain.

Overall, CTRCD occurred in 179 patients (21.1%). Most cases were classified as mild, whereas moderate and severe dysfunction represented progressively smaller proportions of the total cardiotoxicity burden. Clinically overt heart failure was considerably less frequent than imaging-defined CTRCD, indicating that most events were detected during the asymptomatic or subclinical phase of cardiac dysfunction. During longitudinal surveillance, deterioration in LVEF and LV-GLS generally occurred in parallel, without evidence of a consistent temporal precedence of LV-GLS changes over LVEF deterioration. Notably, when the myocardial distribution of CTRCD could be reliably characterized, cardiac dysfunction was more frequently circumscribed than diffuse, with predominant involvement of the inferior and inferoseptal myocardial territories, particularly at the basal or mid-basal level. Conversely, global or diffuse LV dysfunction and mixed patterns combining global impairment with inferior/inferoseptal predominance were less frequently observed. These findings indicate that anti-HER2-related myocardial dysfunction in our cohort frequently manifested as a localized regional abnormality rather than as homogeneous impairment of the entire LV myocardium, as illustrated by the representative LV speckle-tracking bull’s-eye plots shown in Figure 1.

### 3.4. Management and Recovery of Cardiac Dysfunction

Development of CTRCD resulted in temporary or permanent interruption of oncological therapy in a minority of patients. These decisions were primarily driven by the magnitude of LVEF deterioration, particularly when LVEF declined by ≥10 percentage points and/or fell below 50%, the concomitant deterioration in LV-GLS (>15% relative decline from baseline), and, in the more clinically relevant cases, the occurrence of heart failure symptoms and/or biomarker elevation. Treatment decisions were individualized according to the overall severity of cardiac dysfunction and the clinical and oncological context.

Conversely, initiation or intensification of cardioprotective treatment was frequent, occurring in 139 of the 179 patients who developed CTRCD (77.7%). Cardioprotective therapy was not systematically initiated in every patient meeting CTRCD criteria. This may partly reflect the predominance of mild, asymptomatic events, which could be identified on the basis of subclinical LV-GLS deterioration and/or biomarker elevation despite preserved LVEF. In this retrospective real-world cohort, therapeutic decisions were individualized by the treating physicians according to the severity and clinical expression of CTRCD, changes in LVEF and LV-GLS, biomarker findings when available, concomitant cardiovascular conditions, and the overall oncological context. The retrospective dataset did not permit reliable reconstruction of the specific reason for withholding cardioprotective therapy in each untreated patient. Cardioprotective treatment most commonly involved beta-blockers and renin–angiotensin system inhibitors, with mineralocorticoid receptor antagonists, diuretics, and SGLT2 inhibitors less frequently used according to the clinical setting (Table 7).

LV systolic dysfunction showed a predominantly favorable course after recognition and management of cardiotoxicity. Complete LVEF recovery was documented in 124 of 179 patients with CTRCD (69.3%), while partial recovery occurred in 30 patients (16.8%). No documented recovery was observed in 15 patients (8.4%), whereas recovery status was unavailable in 10 patients (5.6%). Overall, complete or partial LVEF recovery was documented in 154 of 179 patients (86.0%), supporting the predominantly reversible course of cardiac dysfunction observed during structured cardio-oncology surveillance.

### 3.5. Oncological Outcomes

At the latest available oncological assessment, approximately half of the cohort was free from evidence of active disease. Among patients with metastatic cancer, a substantial proportion achieved complete or partial response or maintained stable disease, whereas a smaller group experienced progressive disease or entered a palliative phase. A further subgroup was still receiving neoadjuvant or adjuvant treatment with curative intent at the end of the observation period (Table 8).

These categories describe oncological status at the latest documented clinical assessment and should not be interpreted as survival or mortality outcomes.

### 3.6. Clinical and Treatment-Related Factors Associated with CTRCD

Univariable logistic regression analysis was performed to investigate the association of clinical, oncological, treatment-related, and baseline echocardiographic variables with the occurrence of CTRCD (Table 9).

Among conventional cardiovascular risk factors, age, hypertension, diabetes mellitus, dyslipidemia, and current or previous smoking were not significantly associated with CTRCD. BMI showed a borderline association, although statistical significance was not reached. Similarly, neither tumor grade, nor previous anthracycline exposure, nor the number of anthracycline cycles were associated with a significantly increased risk of cardiac dysfunction. Baseline echocardiographic measures, including LVEF and LV-GLS, were also not significantly associated with subsequent CTRCD.

Conversely, overall comorbidity burden and cumulative anti-HER2 exposure emerged as the two main factors associated with CTRCD. Increasing CCI was associated with progressively higher odds of cardiac dysfunction, with an OR of 1.32 (95% CI 1.10–1.58; *p* = 0.003) for each one-point increase. A similar association was observed for cumulative anti-HER2 treatment, with an OR of 1.37 (95% CI 1.09–1.71; *p* = 0.006) for every five additional treatment cycles. Notably, this association was observed for cumulative anti-HER2 exposure rather than for previous anthracycline treatment, suggesting that the duration of HER2-targeted therapy may provide additional information when evaluating treatment-related cardiac risk.

A parsimonious multivariable logistic regression model was subsequently constructed including CCI and cumulative anti-HER2 exposure (Table 10).

With 179 CTRCD events and two covariates included in the final model, the events-per-variable ratio was 89.5. Thus, the restriction to two variables was not imposed by limited statistical power or by a formal events-per-variable constraint, but reflected the exploratory objective of the analysis and the decision to focus on the two variables showing the strongest associations with CTRCD in univariable analysis. The model was therefore intended to assess their mutually adjusted associations with CTRCD rather than to provide a comprehensive multivariable prediction model.

Both variables remained associated with CTRCD after mutual adjustment. Each one-point increase in CCI was associated with a 35% increase in the odds of CTRCD (adjusted OR 1.35, 95% CI 1.12–1.63; *p* = 0.002), while every five additional anti-HER2 cycles were associated with a 41% increase in the odds of CTRCD (adjusted OR 1.41, 95% CI 1.10–1.80; *p* = 0.006). These findings indicate that both comorbidity burden and cumulative treatment exposure retained associations with CTRCD when considered simultaneously; however, the model was not intended to establish their independence from all potential clinical or oncological confounders.

### 3.7. CTRCD Across Increasing Comorbidity Burden and Anti-HER2 Exposure

To further characterize these associations and facilitate their clinical interpretation, observed CTRCD proportions were examined across increasing levels of CCI and cumulative anti-HER2 exposure.

**Figure 2:**
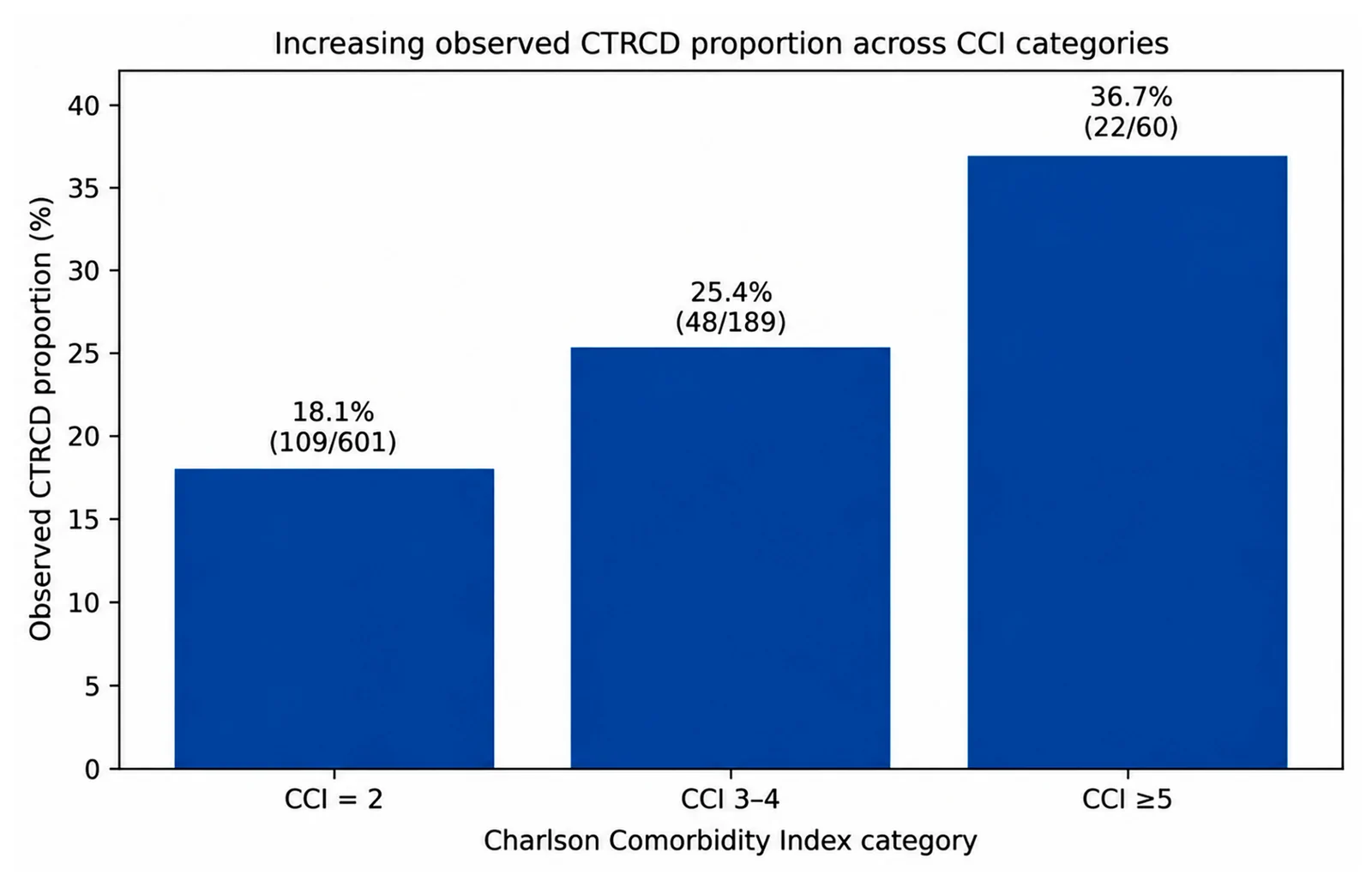
Observed proportion of cancer-therapy-related cardiac dysfunction across increasing Charlson Comorbidity Index categories. Percentages represent the observed proportion of patients developing CTRCD within each CCI category. CCI, Charlson Comorbidity Index; CTRCD, cancer-therapy-related cardiac dysfunction.

A clear graded relationship emerged across CCI categories (Figure 2).

The observed proportion of CTRCD was lowest among patients with CCI = 2 (18.1%; 109/601), increased to 25.4% (48/189) among those with CCI 3–4, and reached 36.7% (22/60) in patients with CCI ≥5. Thus, patients in the highest comorbidity category showed approximately twice the observed proportion of CTRCD compared with those with the lowest CCI burden. The progressive rather than abrupt increase across categories was consistent with the continuous association observed in logistic regression and suggests that the relationship between comorbidity burden and cardiac vulnerability extends across the spectrum of CCI rather than being confined to patients with very high scores.

**Figure 3:**
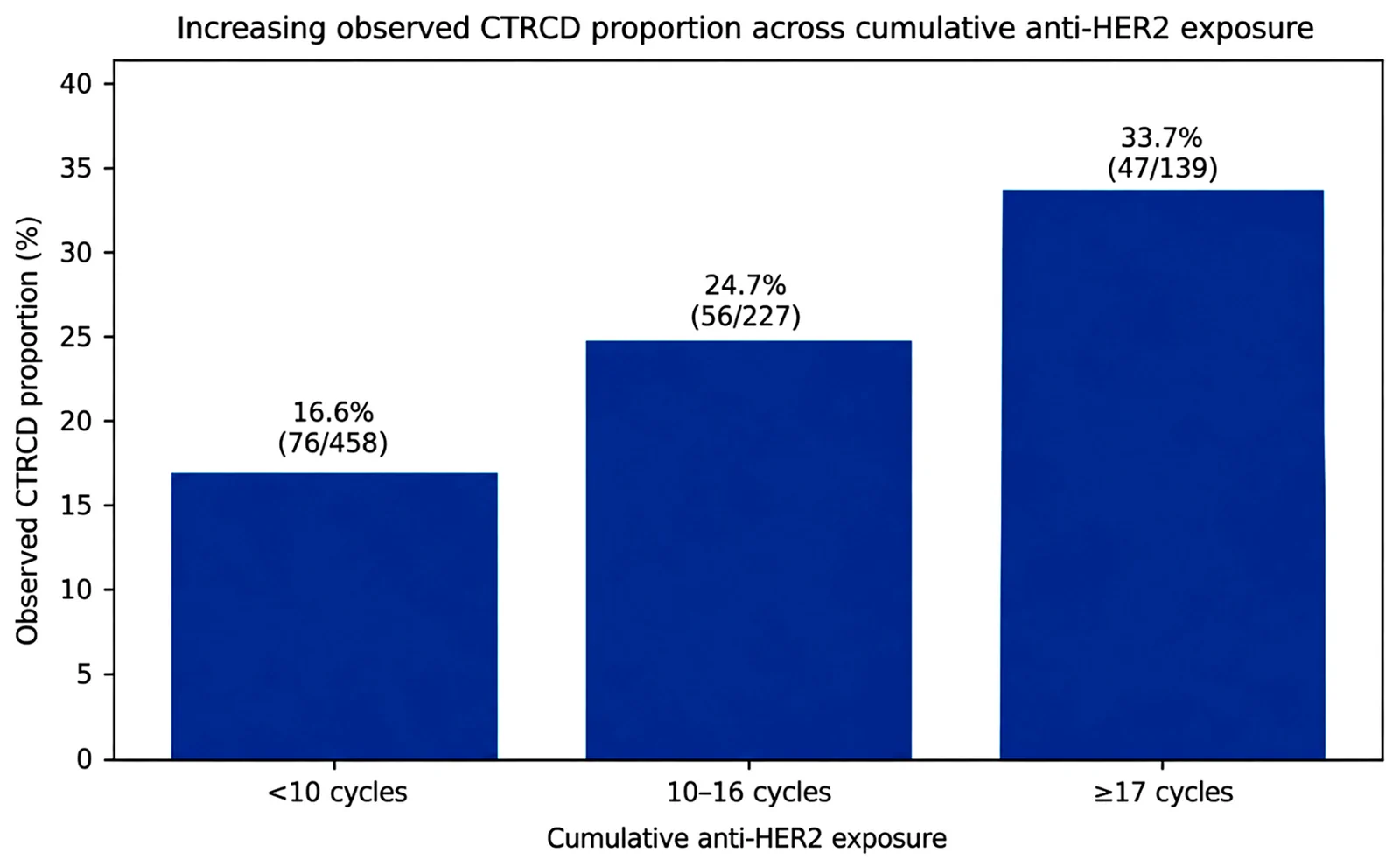
Observed proportion of cancer-therapy-related cardiac dysfunction across increasing cumulative anti-HER2 exposure categories. Percentages represent the observed proportion of patients developing CTRCD within each cumulative anti-HER2 exposure category. CTRCD, cancer-therapy-related cardiac dysfunction; HER2, human epidermal growth factor receptor 2.

A similarly progressive pattern was observed according to cumulative anti-HER2 treatment exposure (Figure 3).

The observed proportion of CTRCD increased from 16.6% among patients exposed to <10 cycles, to 24.7% among those receiving 10–16 cycles, and to 33.7% among those receiving ≥17 cycles. The near-doubling in the observed proportion of CTRCD between the lowest and highest exposure categories was consistent with the association observed when cumulative anti-HER2 exposure was modeled as a continuous variable, for which each five-cycle increment was associated with higher odds of CTRCD.

Taken together, the two analyses showed parallel gradients in the observed proportion of CTRCD, with progressively higher proportions observed both with greater baseline comorbidity burden and with longer cumulative anti-HER2 exposure. These findings complement the multivariable analysis and suggest that assessment of cardiotoxic risk may benefit from considering both the clinical vulnerability of the patient before treatment and the cumulative therapeutic burden accrued during the oncological pathway.

## 4. Discussion

### 4.1. Main Findings

The present study provides a comprehensive characterization of CTRCD in a large cohort of patients with breast cancer exposed to HER2-targeted therapy undergoing longitudinal cardiovascular surveillance within a dedicated cardio-oncology program over a 10-year accrual period. The findings provide a real-world perspective on the frequency, clinical expression, echocardiographic evolution, management, and reversibility of cardiac dysfunction associated with anti-HER2 treatment. CTRCD occurred in approximately one-fifth of the study population and was predominantly represented by mild forms of cardiac dysfunction, whereas moderate and severe events were less frequent and clinically overt heart failure occurred only in a minority of patients. Importantly, when the myocardial distribution of dysfunction could be characterized, anti-HER2-related myocardial impairment more frequently exhibited a regional rather than diffuse pattern, with preferential involvement of the inferior and inferoseptal segments, particularly at the basal and mid-basal levels. This distribution emphasizes the importance of systematic cardiac surveillance during HER2-targeted therapy, since a substantial proportion of cardiotoxic events may be identified at an asymptomatic or subclinical stage, before progression to overt heart failure.

The longitudinal echocardiographic findings further support this concept. LVEF and LV-GLS showed a parallel deterioration during treatment, reaching their lowest values at nadir, followed by substantial improvement toward baseline levels at subsequent follow-up. Together with the high proportion of patients showing recovery of LV systolic function after CTRCD, with generally concordant improvement in LVEF and LV-GLS, this pattern is consistent with the potentially reversible phenotype traditionally associated with HER2-related myocardial dysfunction, particularly when cardiac abnormalities are recognized early and appropriate cardiovascular management is implemented. Cardioprotective therapy was frequently initiated or intensified after detection of CTRCD, whereas interruption of oncological treatment was required in a considerably smaller proportion of the overall cohort. These observations are clinically relevant because contemporary cardio-oncology management should aim not only to prevent progression of myocardial dysfunction, but also to preserve the possibility of continuing effective anticancer treatment whenever safely feasible.

A particularly relevant finding of the present analysis concerns the factors associated with CTRCD. Conventional cardiovascular risk factors considered individually, including age, hypertension, diabetes mellitus, dyslipidemia, and smoking, were not significantly associated with cardiac dysfunction. Likewise, previous anthracycline exposure, anthracycline treatment burden, tumor grade, and baseline measures of LV systolic function were not significantly associated with CTRCD. Conversely, global comorbidity burden, quantified by the CCI, and cumulative anti-HER2 treatment exposure remained associated with CTRCD after mutual adjustment. Each one-point increase in CCI was associated with higher odds of CTRCD, while increasing cumulative anti-HER2 exposure showed a parallel association when evaluated per five additional treatment cycles. Both variables retained their associations after mutual adjustment in the parsimonious multivariable model.

These findings suggest that susceptibility to anti-HER2-related cardiac dysfunction may reflect the interaction between patient-related vulnerability and treatment-related burden rather than isolated cardiovascular risk factors. The CCI may capture reduced physiological reserve by integrating cardiovascular and non-cardiovascular comorbidities, while cumulative anti-HER2 exposure may provide a dynamic measure of treatment-related burden, particularly during prolonged or multiple lines of therapy.

The progressive increase in observed CTRCD proportion across higher CCI categories and longer anti-HER2 exposure further supports this concept. These parallel gradients suggest that cardiotoxic risk is not a fixed baseline characteristic but may evolve throughout treatment. Accordingly, integrating baseline comorbidity burden and cumulative anti-HER2 exposure with conventional clinical and echocardiographic assessment may help identify patients requiring closer cardiovascular surveillance during prolonged HER2-targeted therapy.

### 4.2. Comparison with Previous Evidence on Anti-HER2-Related Cardiotoxicity

The incidence of CTRCD observed in the present study is broadly consistent with, although somewhat higher than, that reported in several contemporary cohorts of patients receiving trastuzumab-based therapy. Differences across studies are expected, since the reported frequency of anti-HER2-related cardiotoxicity is highly dependent on patient selection, previous exposure to cardiotoxic treatments, duration of HER2-targeted therapy, frequency of cardiac surveillance, and, importantly, the definition used to identify cardiac dysfunction [48,49,50]. In a large real-world cohort of 931 patients receiving trastuzumab for HER2-positive early breast cancer, cardiotoxicity was reported in 16.6% of patients, whereas an LVEF decline ≥10 percentage points occurred in 15.1% and LVEF < 50% in 5.9%. Symptomatic heart failure was substantially less frequent [51]. These findings closely resemble the pattern observed in our population, in which CTRCD was identified in 21.1%, an LVEF decline ≥ 10 percentage points occurred in 17.1%, LVEF < 50% at nadir in 5.9%, and clinically overt heart failure in only 2.9%.

The slightly higher overall CTRCD rate in our cohort may be explained, at least in part, by the broader contemporary definition of cardiac dysfunction adopted in the present study. Current cardio-oncology criteria incorporate not only clinically meaningful reductions in LVEF but also changes in myocardial deformation and cardiac biomarkers, thereby permitting recognition of mild asymptomatic CTRCD before conventional systolic impairment becomes evident. The ESC cardio-oncology framework specifically distinguishes mild, moderate, and severe asymptomatic CTRCD and emphasizes the integration of symptoms, LVEF, GLS, and biomarkers in patients receiving HER2-targeted therapy [25]. Accordingly, direct comparison of overall CTRCD incidence with historical studies based predominantly on LVEF decline or symptomatic heart failure should be interpreted cautiously. In our population, the predominance of mild CTRCD and the relatively low occurrence of clinical heart failure support the concept that structured surveillance shifts detection toward earlier stages of myocardial dysfunction. An additional finding was the predominantly regional rather than diffuse distribution of myocardial dysfunction, with preferential involvement of the inferior and inferoseptal territories, particularly at the mid-basal level. Previous trastuzumab studies have largely focused on changes in global LV-GLS, demonstrating that strain deterioration may precede LVEF decline and provide an early marker of CTRCD, whereas the segmental distribution of myocardial impairment has been less extensively characterized [52,53,54]. Interestingly, MVP/billowing was the most frequently documented pre-existing cardiac phenotype in our cohort. A previous study has shown that MVP may be associated with preferential impairment of basal myocardial deformation, potentially related to mechanical traction exerted by the mitral apparatus and, in some individuals, to extrinsic compressive effects associated with a reduced antero-posterior thoracic diameter or anterior chest wall deformity [55]. These observations raise the hypothesis that basal inferior and inferoseptal myocardial territories may represent mechanically more vulnerable regions in some patients with MVP, potentially increasing their susceptibility to additional functional impairment during HER2-targeted therapy. Our findings therefore suggest that early anti-HER2-related dysfunction may not necessarily involve the LV myocardium homogeneously and highlight the potential value of considering regional strain patterns in addition to global LV-GLS.

Previous evidence has also consistently demonstrated that the cardiovascular safety profile of HER2-targeted therapy is strongly influenced by the characteristics of the population under investigation. Clinical trials generally report relatively low rates of severe cardiac events, whereas observational cohorts—including older patients and individuals with cardiovascular comorbidities or reduced cardiac reserve—have reported substantially greater cardiotoxicity [56,57,58,59,60]. This distinction is relevant to our study because the population was derived from routine cardio-oncology practice and included patients with heterogeneous cardiovascular profiles, previous anthracycline exposure, metastatic disease, and variable durations of HER2-targeted treatment. Approximately one-third had pre-existing cardiac disease, while the CCI identified a subgroup with substantial global comorbidity burden. Such heterogeneity arguably reflects the clinical population encountered in contemporary cardio-oncology more closely than highly selected registration trials.

Our findings concerning the clinical course of CTRCD are also consistent with previous observations, suggesting that trastuzumab-related myocardial dysfunction is frequently, although not invariably, reversible [61,62]. In a real-world cohort of patients with metastatic HER2-positive breast cancer and impaired baseline LVEF, most patients developing severe cardiotoxicity subsequently experienced either complete or partial recovery [63]. Similarly, studies evaluating a permissive cardiotoxicity strategy have shown that many patients recover LV function despite continuation or subsequent completion of trastuzumab, although persistent dysfunction may remain in a minority at longer-term follow-up [64,65]. In our cohort, complete LVEF recovery was considerably more frequent than partial or absent recovery, while oncological treatment interruption for cardiotoxicity was required in only a minority of patients. These observations are aligned with contemporary ESC recommendations supporting continuation of HER2-targeted therapy, accompanied by cardioprotective treatment and intensified surveillance, in appropriately selected patients with asymptomatic moderate CTRCD, rather than systematic treatment withdrawal [25].

The comparison with the previous literature becomes particularly relevant when treatment duration is considered. Evidence from randomized trials and meta-analyses has suggested that longer trastuzumab exposure may carry a greater cardiac burden. A meta-analysis including more than 11,000 patients showed that 12 months of trastuzumab was associated with higher odds of clinically relevant cardiac dysfunction than shorter treatment schedules, including a significant excess risk when 12 months was compared specifically with 6 months [66]. Earlier evidence similarly suggested that extending trastuzumab treatment beyond the conventional duration did not improve oncological efficacy while increasing cardiotoxicity [67,68]. These observations provide an important context for our finding that cumulative anti-HER2 exposure, when considered according to the number of administered cycles, was associated with CTRCD. Rather than considering HER2-targeted treatment as a simple binary exposure, our results therefore extend previous evidence by supporting the potential relevance of cumulative treatment burden within an entirely anti-HER2-treated population.

At the same time, our findings differ in part from previous reports regarding conventional determinants of trastuzumab cardiotoxicity. Advanced age, cardiovascular risk factors, reduced baseline LVEF, and particularly previous anthracycline exposure have frequently been associated with increased risk of cardiac dysfunction [69,70,71,72]. In the present analysis, however, these individual variables were not significantly associated with CTRCD, whereas overall comorbidity burden quantified by the CCI did. This apparent discrepancy may be clinically informative rather than contradictory. It raises the possibility that, in a heterogeneous real-world population universally exposed to anti-HER2 treatment, the aggregate burden of coexisting disease may provide information not fully captured by individual cardiovascular risk factors considered separately. This hypothesis is supported by previous real-world evidence showing progressively higher trastuzumab cardiotoxicity across increasing baseline cardiovascular risk categories. The specific potential role of CCI as an integrated marker of susceptibility to anti-HER2-related CTRCD therefore represents one of the most distinctive findings of the present study and deserves separate consideration.

### 4.3. Pathophysiological Considerations

The association between increasing comorbidity burden, cumulative anti-HER2 exposure, and CTRCD may be interpreted within a pathophysiological framework in which myocardial susceptibility reflects the balance between pre-existing cardiovascular reserve and treatment-related stress. HER2 signaling has an important role in cardiomyocyte survival, adaptive responses, and maintenance of myocardial homeostasis, particularly under conditions of cellular stress [73,74]. Pharmacological inhibition of this pathway may therefore reduce the ability of cardiomyocytes to tolerate additional hemodynamic, metabolic, inflammatory, or oxidative insults [75,76,77]. Unlike the cumulative structural myocardial injury classically associated with anthracyclines, HER2-related dysfunction has traditionally been considered predominantly functional and potentially reversible [78]. However, the clinical expression of this toxicity is heterogeneous, suggesting that the myocardial response to HER2 inhibition is substantially influenced by the biological substrate on which treatment acts.

The association observed with CCI supports this concept. A higher CCI should not necessarily be interpreted as the effect of any single comorbid condition, but rather as a surrogate of reduced global physiological reserve. Cardiovascular disease, diabetes, renal dysfunction, pulmonary disease, metabolic abnormalities, and other chronic conditions may converge through endothelial dysfunction, neurohormonal activation, systemic inflammation, oxidative stress, impaired mitochondrial function, and reduced capacity for cardiovascular adaptation [79,80,81]. In this setting, inhibition of HER2-dependent cardioprotective signaling may represent an additional stressor capable of revealing previously compensated myocardial vulnerability. This interpretation may also explain why individual conventional cardiovascular risk factors were not independently associated with CTRCD in our analysis, whereas their broader clinical context, captured by an integrated comorbidity index, provided greater discriminatory information. The CCI may therefore reflect a multidimensional susceptibility phenotype that is not adequately represented by evaluating hypertension, diabetes, dyslipidemia, or smoking separately.

A complementary mechanism may underlie the association with cumulative anti-HER2 exposure. Repeated treatment cycles result in prolonged inhibition of cardiomyocyte HER2 signaling and may progressively reduce the capacity of the myocardium to compensate for concurrent biological and hemodynamic stress [82,83]. The observed relationship should not necessarily be interpreted as evidence of irreversible dose-dependent myocardial injury analogous to anthracycline toxicity. Rather, longer exposure may increase the temporal opportunity for cardiac dysfunction to emerge, particularly in patients with limited cardiovascular reserve or concomitant exposure to other cardiotoxic treatments. The progressive increase in observed CTRCD proportion across longer treatment-duration categories is consistent with this concept and suggests that cardiotoxic risk may evolve during therapy rather than remaining determined exclusively by baseline characteristics [84].

The longitudinal echocardiographic course further supports a predominantly functional and potentially reversible process, with LVEF and LV-GLS improving toward baseline after deterioration at nadir and complete recovery being more frequent than persistent LV dysfunction. Increasing cumulative exposure may therefore identify a period of greater myocardial vulnerability without necessarily implying permanent damage. Patients with greater comorbidity burden may reach the threshold for functional myocardial impairment earlier during treatment, supporting a dynamic interaction between host vulnerability, myocardial reserve, and cumulative anti-HER2 exposure.

### 4.4. Clinical Implications

The present findings may have relevant implications for cardiovascular risk assessment and surveillance in patients receiving HER2-targeted therapy. Contemporary cardio-oncology strategies rely heavily on baseline cardiovascular evaluation followed by serial assessment of LVEF, LV-GLS, and cardiac biomarkers. Although this approach remains essential, our results suggest that longitudinal cardiovascular risk assessment may potentially be informed by considering two readily obtainable and complementary dimensions: baseline global comorbidity burden and cumulative anti-HER2 exposure. Rather than considering cardiotoxic risk as a static characteristic established before treatment, these findings support a more dynamic model in which baseline susceptibility interacts with the progressive therapeutic burden accumulated during the oncological pathway.

The CCI may be particularly useful in this context because it provides a simple means of summarizing multiple coexisting diseases that, when considered individually, may have limited predictive value. In the present population, conventional cardiovascular risk factors did not independently identify patients at increased risk of CTRCD, whereas increasing CCI remained associated with cardiac dysfunction after adjustment for cumulative anti-HER2 exposure. The progressive increase in CTRCD across CCI categories further suggests that comorbidity burden should be regarded as a continuum rather than a binary high- versus low-risk condition. From a practical perspective, calculation of CCI at the initial cardio-oncology assessment is inexpensive, rapid, reproducible, and based entirely on routinely available clinical information. It could therefore complement, rather than replace, established cardiovascular risk-assessment tools and imaging parameters.

The association with cumulative anti-HER2 exposure has a different but equally relevant clinical implication. Unlike baseline comorbidity, treatment exposure is time-dependent and progressively accumulates during therapy. A patient initially considered at relatively low cardiovascular risk may therefore require reassessment as the number of anti-HER2 cycles increases, particularly when treatment extends beyond conventional schedules because of maintenance therapy, recurrence, or metastatic disease. The progressive increase in CTRCD across the <10, 10–16, and ≥17-cycle categories supports the potential usefulness of incorporating cumulative exposure into surveillance planning. Importantly, these thresholds should not presently be interpreted as rigid cut-offs for treatment interruption. Rather, they may identify clinically intuitive stages at which cardiovascular risk should be reconsidered and surveillance intensity potentially adapted to the evolving risk profile.

The combination of these two dimensions may be particularly informative. Patients with higher CCI and prolonged anti-HER2 exposure could represent a subgroup in whom closer echocardiographic surveillance, greater attention to changes in LV-GLS and biomarkers, aggressive control of modifiable cardiovascular risk factors, and a lower threshold for initiation of cardioprotective therapy may be appropriate. Conversely, patients with a low comorbidity burden and limited cumulative exposure might potentially avoid unnecessarily intensive surveillance, although this possibility requires prospective validation before influencing clinical practice. The objective should therefore not be to use CCI or treatment duration as isolated reasons to withhold effective oncological therapy, but to improve identification of patients in whom the balance between oncological benefit and cardiovascular risk warrants closer multidisciplinary attention.

**Figure 4:**
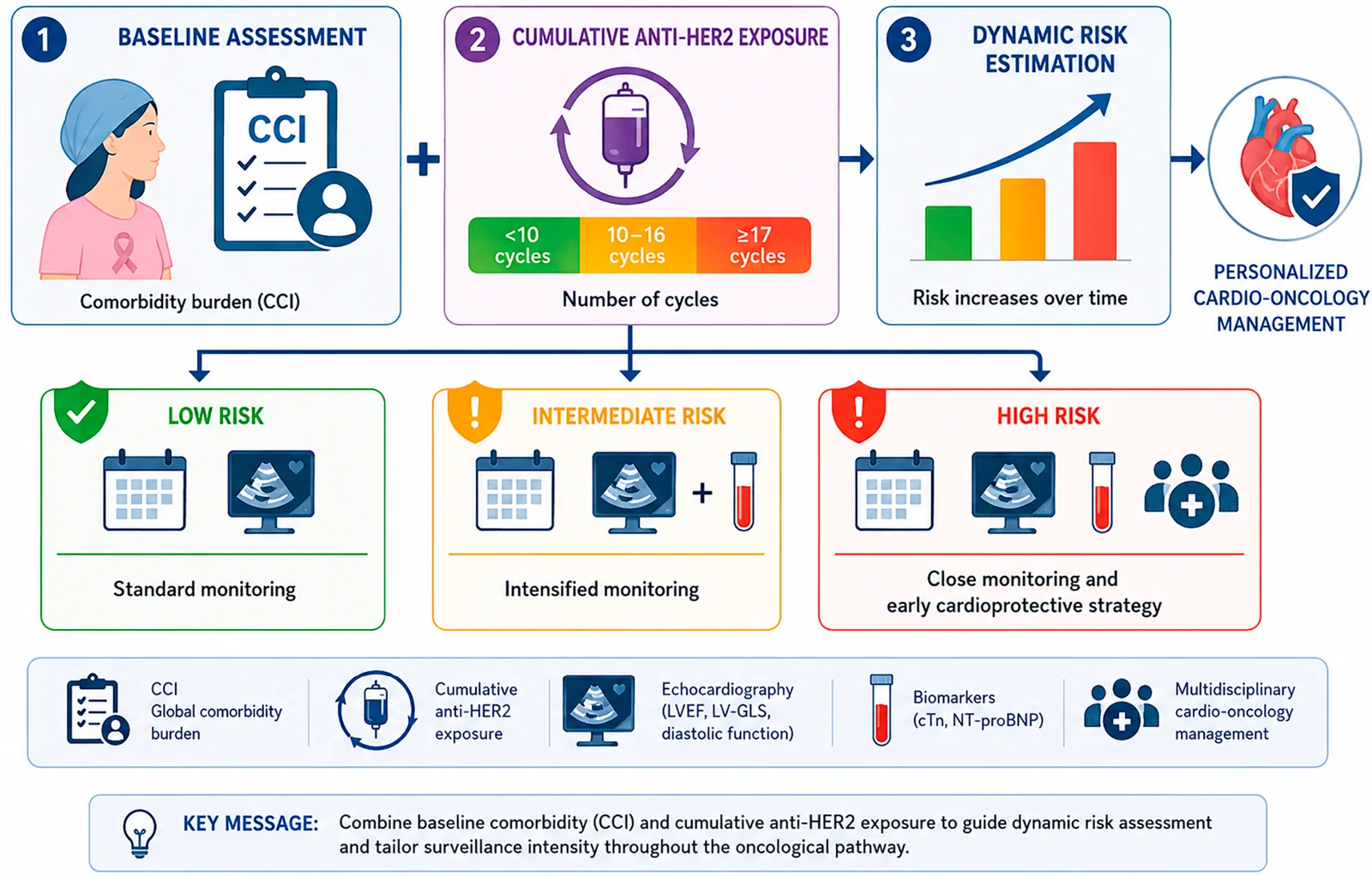
Proposed framework for dynamic cardiovascular risk assessment and surveillance during HER2-targeted therapy. The framework integrates baseline global comorbidity burden, assessed by the Charlson Comorbidity Index (CCI), with longitudinal evaluation of cumulative anti-HER2 exposure. Increasing comorbidity burden and cumulative treatment exposure may identify patients requiring progressively intensified cardiovascular surveillance, ranging from standard monitoring to closer echocardiographic and biomarker assessment, early cardioprotective strategies, and multidisciplinary cardio-oncology management. This approach emphasizes dynamic reassessment of cardiovascular risk throughout the oncological pathway rather than reliance on baseline risk stratification alone. The proposed framework is hypothesis-generating and requires prospective validation before implementation in routine clinical practice. CCI, Charlson Comorbidity Index; cTn, cardiac troponin; HER2, human epidermal growth factor receptor 2; LV-GLS, left ventricular global longitudinal strain; LVEF, left ventricular ejection fraction; NT-proBNP, N-terminal pro-B-type natriuretic peptide.

A hypothesis-generating framework integrating preliminary CCI assessment with longitudinal consideration of cumulative anti-HER2 exposure as potential complementary information for dynamic cardiovascular risk reassessment is summarized in Figure 4. 

Most CTRCD events were mild, clinical heart failure was uncommon, and LV systolic recovery was frequently observed, while interruption of oncological treatment for cardiotoxicity occurred in only a minority of patients. These observations reinforce the contemporary principle that detection of CTRCD should not automatically lead to permanent discontinuation of HER2-targeted therapy [85]. When clinically appropriate, early recognition of myocardial dysfunction, prompt cardioprotective treatment, intensified surveillance, and close collaboration between cardiologists and oncologists may allow continuation or resumption of an effective anticancer treatment while limiting progression toward symptomatic or irreversible cardiac dysfunction.

Taken together, these findings suggest a pragmatic evolution from baseline risk stratification toward longitudinal risk reassessment during HER2-targeted treatment. A patient’s cardiovascular risk profile at the beginning of therapy represents only the starting point; comorbidity burden defines the underlying substrate, while cumulative treatment exposure progressively modifies risk throughout follow-up. If prospectively validated, integrating these readily accessible variables with conventional echocardiographic and biomarker surveillance could contribute to a more individualized cardio-oncology pathway, directing resources and monitoring intensity toward patients who are most likely to develop CTRCD while preserving effective HER2-targeted therapy whenever safely possible.

Finally, emerging evidence suggests a potential cardioprotective role for SGLT2 inhibitors in patients receiving cardiotoxic cancer therapies, although their efficacy in this setting requires confirmation in randomized clinical trials [86].

### 4.5. Strengths and Limitations

The present study has several strengths, including the relatively large sample size, the extended accrual period, and the specific focus on patients with breast cancer exposed to HER2-targeted therapy in a real-world cardio-oncology setting. The availability of longitudinal echocardiographic examinations allowed cardiac function to be evaluated at baseline, at nadir, and during subsequent follow-up rather than relying on a single assessment. Moreover, most echocardiographic examinations were performed by the same experienced cardiologist, while all STE analyses were performed offline by a single operator using the same software platform, thereby limiting variability related to image interpretation and post-processing. The simultaneous evaluation of conventional cardiovascular risk factors, global comorbidity burden, previous anthracycline exposure, cumulative anti-HER2 treatment, and echocardiographic parameters also permitted a multidimensional assessment of factors potentially associated with CTRCD.

Several limitations should nevertheless be acknowledged. The retrospective, single-center design inherently exposes the study to selection bias, incomplete documentation, and residual or unmeasured confounding, and limits the generalizability of the findings to other cardio-oncology populations and healthcare settings. The cohort was almost exclusively composed of women with breast cancer, reflecting the population most commonly receiving HER2-targeted therapy but limiting the generalizability of our findings to male patients with breast cancer and to patients receiving anti-HER2 agents for other malignancies. Furthermore, treatment strategies were heterogeneous and included different combinations and sequences of HER2-targeted agents, anthracyclines, taxanes, radiotherapy, and endocrine therapy. Consequently, the independent contribution of individual therapeutic components cannot be completely disentangled from the overall treatment pathway.

The long observation period represents both a strength and a potential source of heterogeneity. During the decade covered by the study, oncological strategies, indications for HER2-targeted agents, cardiovascular surveillance protocols, and management of CTRCD evolved considerably. In addition, the timing and number of echocardiographic examinations were determined by routine clinical practice rather than by a uniform research schedule. Consequently, the exact timing of first CTRCD occurrence could not be reliably determined for all affected patients, precluding robust estimation of time to CTRCD.

Although most examinations were performed by the same operator and all strain analyses were centralized, adequate images for retrospective STE analysis were not available at every time point in all patients. Mild asymptomatic CTRCD may be identified despite preserved LVEF on the basis of a >15% relative decline in LV-GLS and/or cardiac biomarker elevation; therefore, incomplete availability of these measurements may have resulted in under-ascertainment or misclassification of mild CTRCD. Missing LV-GLS or biomarker values were not considered normal, but patients without these measurements had fewer opportunities to fulfill mild CTRCD criteria. Moreover, as measurement availability was determined by routine clinical practice and may not have been random, differential misclassification cannot be excluded. Accordingly, the reported frequency of mild CTRCD should be interpreted with appropriate caution.

Furthermore, although initiation or intensification of cardioprotective therapy was documented, the exact duration of treatment, subsequent dose titration, temporary discontinuation, and continuation after recovery of LV systolic function could not be reliably reconstructed for all treated patients. Accordingly, no standardized duration of cardioprotective treatment can be reported, and the observed recovery of LV systolic function should not be interpreted as demonstrating an effect attributable to a specific duration of cardioprotective therapy.

In addition, complete longitudinal ascertainment of vital status and cause-specific mortality was not available for the entire cohort. Therefore, the oncological categories reported at the latest available follow-up should not be interpreted as survival outcomes, and robust estimates of overall, cancer-related, cardiovascular, and other-cause mortality could not be provided.

The intrinsic methodological limitations of both LVEF and GLS assessment should also be acknowledged, particularly because relatively small longitudinal changes in these parameters may influence the diagnosis and grading of CTRCD in cardio-oncology practice. LVEF assessment is dependent on adequate image quality and endocardial border definition, geometric assumptions, loading conditions, and operator experience, and is characterized by non-negligible intra- and inter-observer variability and limited sensitivity for detecting subtle changes in LV systolic function [87,88,89,90]. Although GLS may provide greater sensitivity for identifying subclinical myocardial dysfunction, STE-derived measurements are themselves influenced by image quality, frame rate, tracking quality, loading conditions, operator-related factors, and inter-vendor/software variability [91,92,93,94]. Furthermore, extrinsic anthropometric factors, particularly chest wall conformation and a reduced antero-posterior thoracic diameter, may affect cardiac motion and the reproducibility of both LVEF and GLS measurements [95]. These limitations are especially relevant in cardio-oncology, where serial examinations are used to identify relatively small treatment-related changes from baseline and may consequently affect CTRCD classification and subsequent clinical management. Accordingly, changes in LVEF and GLS observed during longitudinal surveillance should be interpreted within the overall clinical context and, whenever possible, considering acquisition quality, technical consistency, and loading conditions rather than as isolated numerical changes.

The CCI also warrants specific consideration. Although simple, reproducible, and readily applicable, it was originally developed as a prognostic measure of overall comorbidity and was not specifically designed to predict cardiotoxicity. Some of its components may therefore carry different cardiovascular implications, while the same total score can arise from substantially different combinations of underlying diseases. Moreover, because malignancy and metastatic disease contribute to the conventional CCI, part of the score reflects oncological rather than purely cardiovascular or systemic vulnerability. This is particularly relevant because metastatic disease was also associated with greater cumulative anti-HER2 exposure in the present cohort, raising the possibility that CCI and cumulative treatment exposure partly capture overlapping information related to advanced disease status rather than entirely distinct dimensions of cardiotoxic vulnerability. The association observed between CCI and CTRCD should consequently be interpreted as evidence supporting the potential relevance of global comorbidity burden, rather than as validation of CCI as a dedicated cardio-oncology risk score. Because metastatic status was not separately included in the parsimonious multivariable model, residual confounding by disease stage cannot be excluded. Accordingly, CCI and cumulative anti-HER2 exposure should be interpreted as mutually adjusted correlates of CTRCD rather than as independent causal determinants.

Cumulative anti-HER2 exposure also requires cautious interpretation. Treatment duration was quantified according to the number of administered cycles, which provides a pragmatic measure of exposure but does not fully account for differences in drug type, dosing schedule, treatment interruptions, combination regimens, or changes in therapy during recurrent or metastatic disease. Furthermore, longer treatment exposure may partly reflect longer survival, treatment response, or different disease trajectories, creating the possibility of time-dependent and indication-related confounding. In particular, the greater number of cycles administered in the metastatic/maintenance setting may have contributed to the observed association between cumulative exposure and CTRCD. Accordingly, this relationship should not be interpreted as evidence of a direct causal dose–toxicity effect. Similarly, the categorical thresholds of <10, 10–16, and ≥17 cycles were intended to facilitate clinical interpretation of the exposure gradient rather than to define validated cardiotoxicity thresholds.

A time-to-event analysis using Cox proportional hazards regression could not be reliably performed because the exact date of first CTRCD occurrence could not be ascertained for all affected patients from the retrospective dataset. For the same reason, a landmark sensitivity analysis was not considered methodologically reliable, because defining exposure and outcome status at a fixed landmark would have required assumptions regarding the timing of CTRCD that were not supported by the available retrospective data. In several cases, CTRCD could only be localized to the interval between consecutive cardiac assessments rather than assigned to a precise date of onset. Consequently, cumulative anti-HER2 exposure was analyzed as the total number of administered cycles rather than as a time-dependent covariate, and the observed association remains potentially susceptible to time-dependent and reverse-causality bias. In particular, CTRCD itself may influence subsequent treatment exposure through temporary or permanent treatment interruption, whereas patients remaining free of CTRCD have a greater opportunity to accumulate additional treatment cycles. Importantly, the present analysis was not designed to develop or validate a formal prediction model, and no assessment of predictive performance, calibration, discrimination, or external validation was performed. Accordingly, CCI and cumulative anti-HER2 exposure should be interpreted as factors associated with CTRCD rather than as validated predictors. The association between cumulative anti-HER2 exposure and CTRCD should therefore be regarded as exploratory and hypothesis-generating rather than predictive or causal. Prospective studies with precisely time-stamped treatment exposure and CTRCD events are required to evaluate this relationship using appropriate time-to-event models.

Finally, despite adjustment in a parsimonious multivariable model, residual confounding cannot be excluded. The number of covariates was intentionally restricted in accordance with the exploratory, hypothesis-generating objective of the analysis, rather than to develop a comprehensive multivariable prediction model. Potentially relevant interactions—particularly between CCI, previous anthracycline exposure, cumulative anti-HER2 treatment, radiotherapy, and cardioprotective therapy—could not be comprehensively investigated. The findings should therefore be regarded as hypothesis-generating and require confirmation in prospective, multicenter cohorts with standardized imaging and biomarker surveillance, predefined treatment-exposure assessment, and external validation. Such studies will be essential to determine whether integrating comorbidity burden and cumulative anti-HER2 exposure into established cardio-oncology risk models can meaningfully improve prediction of CTRCD and guide individualized surveillance strategies.

## 5. Conclusions

In this large real-world cardio-oncology cohort, CTRCD represented a clinically relevant but predominantly mild and frequently reversible complication. Although approximately one-fifth of patients developed CTRCD, clinically overt heart failure was uncommon, and LV systolic function recovered in most affected patients during subsequent follow-up. These findings reinforce the value of structured cardiovascular surveillance for identifying cardiac dysfunction at an early and potentially manageable stage while preserving effective oncological treatment whenever clinically feasible.

Beyond the characterization of CTRCD itself, the principal finding of the present study was the identification of global comorbidity burden and cumulative anti-HER2 exposure as two complementary factors that remained associated with cardiac dysfunction after mutual adjustment. Increasing CCI may reflect reduced physiological reserve and underlying patient vulnerability, whereas progressively greater anti-HER2 exposure may reflect an accumulating treatment-related burden. Their parallel associations with CTRCD suggest the potential value of considering both baseline patient vulnerability and cumulative treatment exposure when reassessing cardiovascular risk throughout the course of HER2-targeted therapy. Importantly, the association between cumulative anti-HER2 exposure and CTRCD should be considered exploratory and hypothesis-generating rather than causal, because time-dependent and reverse-causality bias cannot be excluded.

If prospectively validated, integration of CCI and cumulative treatment exposure with conventional cardiovascular assessment, cardiac biomarkers, LVEF, and LV-GLS may provide additional information for longitudinal cardiovascular risk assessment and potentially help tailor surveillance intensity.

Future prospective multicenter studies with standardized, time-resolved assessment of treatment exposure and CTRCD occurrence should determine whether this integrated approach provides incremental prognostic or predictive information beyond established risk-assessment strategies and whether it can help guide individualized cardiovascular surveillance.

## Figures and Tables

**Table 1:** Demographic, anthropometric, and cardiovascular risk profile of the 10-year cohort.

Variable	10-Year Cohort (*n* = 850)
Age at start of cancer therapy, years	54.1 ± 11.4
Female sex, *n* (%)	845 (99.4)
Male sex, *n* (%)	5 (0.6)
Weight, kg	63.3 ± 15.7
Height, cm	162.0 ± 6.8
BMI, kg/m^2^	23.6 ± 4.7
Hypertension, *n* (%)	268 (31.6)
Diabetes mellitus, *n* (%)	60 (7.0)
Dyslipidemia, *n* (%)	219 (25.7)
Current/former smoking, *n* (%)	159 (18.7)

Data are presented as mean ± SD or *n* (%). BMI, body mass index; SD, standard deviation.

**Table 2:** Pre-existing cardiac disease, comorbidity profile, and Charlson Comorbidity Index distribution in the 10-year cohort.

Variable	10-Year Cohort (*n* = 850)
Pre-existing cardiac disease, *n* (%)	273 (32.2)
Mitral valve prolapse/billowing ± mitral regurgitation, *n* (%)	149 (17.5)
LV hypertrophy/hypertensive heart disease, *n* (%)	75 (8.8)
More-than-mild aortic valve/aortic disease, *n* (%)	50 (5.8)
Arrhythmia/conduction disorder, *n* (%)	35 (4.1)
Ischemic/atherosclerotic cardiovascular disease, *n* (%)	25 (2.9)
**Most frequent comorbidities**	
Osteopenia/osteoporosis, *n* (%)	373 (43.9)
Thyroid disease, *n* (%)	149 (17.5)
Hepatic steatosis/MASLD/NAFLD, *n* (%)	139 (16.4)
Metastatic disease, *n* (%)	119 (14.0)
Anxiety/depressive disorders, *n* (%)	75 (8.8)
Venous thromboembolism/thrombosis, *n* (%)	65 (7.6)
Renal disease/nephrolithiasis, *n* (%)	65 (7.6)
BRCA1/2 mutation, *n* (%)	60 (7.0)
Anemia, *n* (%)	55 (6.4)
Chronic pulmonary disease, *n* (%)	55 (6.4)
**Charlson Comorbidity Index**	
CCI, median [IQR]	2 (2–3)
CCI = 2, *n* (%)	601 (70.8)
CCI 3–4, *n* (%)	189 (22.2)
CCI ≥ 5, *n* (%)	60 (7.0)

Data are presented as *n* (%) unless otherwise specified. The Charlson Comorbidity Index summarizes the overall burden of clinically relevant comorbidities and is reported both as median [IQR] and according to predefined categories. Individual comorbidities are not mutually exclusive; therefore, percentages do not sum to 100%. BRCA, breast cancer susceptibility gene; CCI, Charlson Comorbidity Index; IQR, interquartile range; LV, left ventricular; MASLD, metabolic dysfunction-associated steatotic liver disease; NAFLD, non-alcoholic fatty liver disease.

**Table 3:** Baseline laboratory characteristics of the 10-year cohort.

Variable	10-Year Cohort (*n* = 850)
Hemoglobin, g/dL	12.1 ± 1.5
NLR	2.40 [1.61–3.52]
RDW, %	13.5 [12.9–15.4]
Leukocytes, ×10^9^/L	5.81 ± 2.23
Creatinine, mg/dL	0.72 ± 0.20
eGFR, mL/min/1.73 m^2^	92.8 ± 17.8
AST, U/L	23 [19–34.5]
ALT, U/L	22 (17–31)
Sodium, mEq/L	141.0 ± 3.3
Potassium, mEq/L	4.18 ± 0.38
Total bilirubin, mg/dL	0.60 [0.47–0.80]
Calcium, mmol/L	2.38 [2.27–2.43]
CRP, mg/dL *	0.35 [0.10–3.50]
Cardiac troponin I (cTnI), ng/mL *	0.015 [0.003–0.120]
NT-proBNP, pg/mL *	110 (35–1200)

Data are presented as mean ± SD or median [IQR], as appropriate. * CRP, conventional cardiac troponin I (cTnI; non-high-sensitivity assay), and NT-proBNP values were available only in a subset of patients. The institutional upper reference limit for cTnI was 0.04 ng/mL. ALT, alanine aminotransferase; AST, aspartate aminotransferase; CRP, C-reactive protein; cTnI, cardiac troponin I; eGFR, estimated glomerular filtration rate; IQR, interquartile range; NLR, neutrophil-to-lymphocyte ratio; NT-proBNP, N-terminal pro-B-type natriuretic peptide; RDW, red cell distribution width; SD, standard deviation.

**Table 4:** Tumor characteristics, histological features, and biomarker profile of the 10-year cohort.

Tumor Characteristic	10-Year Cohort (*n* = 850)
**Tumor laterality**	
Left breast, *n* (%)	413 (48.6)
Right breast, *n* (%)	368 (43.3)
Bilateral breast cancer, *n* (%)	45 (5.3)
Occult breast primary, *n* (%)	15 (1.8)
Breast laterality unavailable/unspecified, *n* (%)	9 (1.1)
**Histological type**	
Invasive ductal/NST carcinoma, *n* (%)	716 (84.2)
Invasive lobular carcinoma, *n* (%)	65 (7.6)
Apocrine differentiation, *n* (%)	25 (2.9)
Metaplastic carcinoma, *n* (%)	15 (1.8)
Other special breast histology, *n* (%)	15 (1.8)
Mixed ductal/lobular carcinoma, *n* (%)	5 (0.6)
Other/unspecified breast histology, *n* (%)	9 (1.1)
**Tumor grading**	
G1, *n* (%)	5 (0.6)
G2, *n* (%)	229 (26.9)
G3/high grade, *n* (%)	547 (64.3)
Grade unavailable/not classifiable, *n* (%)	70 (8.2)
**Hormone-receptor profile**	
ER, %	70 [0–95]
ER-positive (≥1%), *n* (%)	566 (66.6)
PgR, %	10 [0–70]
PgR-positive (≥1%), *n* (%)	477 (56.1)
**HER2 status**	
HER2-positive/amplified, *n* (%)	447 (52.6)
HER2-low (IHC 1+ or 2+/ISH−), *n* (%)	199 (23.4)
HER2-zero/negative, *n* (%)	179 (21.1)
HER2 unavailable/indeterminate, *n* (%)	25 (2.9)
Ki-67, %	32 (22–50)

Data are presented as *n* (%) or median [IQR], as appropriate. Percentages may not sum to 100% because of rounding or unavailable/unclassifiable data. ER, estrogen receptor; HER2, human epidermal growth factor receptor 2; IHC, immunohistochemistry; IQR, interquartile range; ISH, in situ hybridization; NST, no special type; PgR, progesterone receptor.

**Table 5:** Anticancer treatment characteristics and therapeutic exposures in the 10-year cohort.

Treatment Variable	10-Year Cohort (*n* = 850)
Neoadjuvant systemic therapy, *n* (%)	249 (29.2)
Anthracycline exposure, *n* (%)	661 (77.8)
AC/EC cycles among exposed, median [IQR]	4 [4–4]
Taxane exposure, *n* (%)	775 (91.2)
Any anti-HER2 therapy, *n* (%)	850 (100)
Trastuzumab exposure, *n* (%)	700 (82.4)
Pertuzumab exposure, *n* (%)	174 (20.5)
T-DM1 (trastuzumab emtansine) exposure, *n* (%)	75 (8.8)
T-DXd (trastuzumab deruxtecan) exposure, *n* (%)	35 (4.1)
Neoadjuvant anti-HER2 therapy, *n* (%)	154 (18.1)
Neoadjuvant anti-HER2 cycles, median [IQR]	4 (4–6)
Adjuvant anti-HER2 therapy, *n* (%)	520 (61.2)
Adjuvant anti-HER2 cycles, median [IQR]	17 (14–17)
Metastatic/maintenance anti-HER2 therapy, *n* (%)	288 (33.9)
Metastatic/maintenance anti-HER2 cycles, median [IQR]	18 (12–30)
Radiotherapy, *n* (%)	646 (76.0)
Endocrine therapy, *n* (%)	502 (59.1)
**Radiotherapy details**	
Breast/chest-wall ± regional nodal RT, *n* (%)	570 (67.1)
Left-sided breast/chest-wall RT, *n* (%)	278 (32.7)
Regional nodal irradiation, *n* (%)	264 (31.1)
Metastatic/palliative or stereotactic RT, *n* (%)	100 (11.8)
Typical locoregional RT dose, Gy	40–50
Hypofractionated RT (15 fractions), *n* (%)	204 (24.0)
Boost to tumor bed, *n* (%)	139 (16.4)
**Endocrine therapy details**	
Aromatase inhibitor-based therapy, *n* (%)	438 (51.5)
Tamoxifen-based therapy, *n* (%)	154 (18.1)
LHRH analogue/ovarian suppression, *n* (%)	109 (12.8)
Fulvestrant exposure, *n* (%)	90 (10.6)

Data are presented as *n* (%), median [IQR], or range, as appropriate. Treatment categories are not mutually exclusive because patients could receive multiple sequential or concomitant anticancer therapies. Individual anti-HER2 agent categories and treatment settings are not mutually exclusive because patients could receive multiple HER2-targeted agents sequentially or in combination across different phases of the oncological treatment course. Anti-HER2 therapy could be administered in the neoadjuvant, adjuvant, and/or metastatic/maintenance setting. Radiotherapy and endocrine therapy subcategories may overlap. AC, doxorubicin plus cyclophosphamide; CDK4/6, cyclin-dependent kinase 4 and 6; EC, epirubicin plus cyclophosphamide; Gy, gray; HER2, human epidermal growth factor receptor 2; IQR, interquartile range; LHRH, luteinizing hormone-releasing hormone; RT, radiotherapy; T-DM1, trastuzumab emtansine; T-DXd, trastuzumab deruxtecan.

**Table 6:** Longitudinal echocardiographic findings, myocardial deformation, and cancer-therapy-related cardiac dysfunction in the 10-year cohort.

Echocardiographic Variable	10-Year Cohort (*n* = 850)
**Cardiac morphology**	
Interventricular septal thickness at baseline, mm	9.6 ± 2.4
LV end-diastolic diameter at baseline, mm	44.3 ± 5.2
Left atrial volume at baseline, mL	47 (40–59)
**LV diastolic function**	
E/A < 1 at baseline, *n* (%)	319 (37.5)
E/A > 1 at baseline, *n* (%)	124 (14.6)
E/A < 1 at final follow-up, *n* (%)	423 (49.8)
E/A > 1 at final follow-up, *n* (%)	204 (24.0)
E/e′ at baseline	7.8 [6.7–9.2]
E/e′ at final follow-up	8.0 [7.0–9.5]
**LV systolic function**	
LVEF at baseline, %	65.2 ± 4.7
LVEF nadir, %	59.2 ± 7.1
LVEF at final follow-up, %	64.2 ± 5.3
Absolute LVEF decline, percentage points	5 [0–10]
LVEF decline ≥ 10 percentage points, *n* (%)	145 (17.1)
LVEF < 50% at nadir, *n* (%)	50 (5.9)
**Myocardial deformation**	
Patients with technically adequate images for longitudinal LV-GLS assessment, *n* (%)	750 (88.2)
LV-GLS not reliably assessable because of inadequate acoustic window/image quality, *n* (%)	100 (11.8)
LV-GLS at baseline, %	19.9 ± 0.8
LV-GLS nadir, %	18.3 ± 1.8
LV-GLS at final follow-up, %	19.4 ± 1.2
Absolute change in LV-GLS from baseline to nadir, percentage points	−1.5 [−3.0–0.0]
Relative LV-GLS decline >15% from baseline, *n* (%)	139 (18.5)
**Pulmonary artery systolic pressure**	
PASP at baseline, mmHg	29 (25–33)
PASP at final follow-up, mmHg	28 (25–32)
**Cancer-therapy-related cardiac dysfunction**	
CTRCD, *n* (%)	179 (21.1)
Mild CTRCD, *n* (%)	119 (14.0)
Moderate CTRCD, *n* (%)	40 (4.7)
Severe CTRCD, *n* (%)	20 (2.4)
Clinical heart failure, *n* (%)	25 (2.9)
**Myocardial pattern/territory in CTRCD**	
Inferior/inferoseptal, predominantly basal or mid-basal involvement, *n* (%)	70 (8.2)
Global/diffuse LV involvement, *n* (%)	25 (2.9)
Mixed global + inferior/inferoseptal involvement, *n* (%)	15 (1.8)
Other/unspecified regional involvement, *n* (%)	10 (1.2)
CTRCD with myocardial territory not documented, *n* (%)	59 (6.9)

Data are presented as mean ± SD, median [IQR], or *n* (%), as appropriate. LV-GLS measurements and derived changes refer to patients with technically adequate images available for longitudinal speckle-tracking analysis (*n* = 750). For ease of interpretation, LV-GLS values are reported as positive absolute values, with lower values indicating worse myocardial deformation. Percentages for CTRCD severity are calculated on the overall cohort. Myocardial patterns describe the predominant distribution of LV involvement among patients developing CTRCD. CTRCD, cancer-therapy-related cardiac dysfunction; E/A, ratio of early (E) to late (A) transmitral flow velocity; E/e′, ratio of early transmitral flow velocity to early diastolic mitral annular velocity; IQR, interquartile range; LV, left ventricular; LV-GLS, left ventricular global longitudinal strain; LVEF, left ventricular ejection fraction; PASP, pulmonary artery systolic pressure; SD, standard deviation.

**Table 7:** Management of CTRCD and subsequent recovery of left ventricular systolic function among patients who developed CTRCD in the 10-year cohort.

Variable	Patients with CTRCD (*n* = 179)
CTRCD management	
Temporary/permanent interruption of oncological therapy for cardiotoxicity, *n*/N (%)	50/179 (27.9)
Cardioprotective/HF therapy initiated for CTRCD, *n*/N (%)	139/179 (77.7)
ACE-i/ARB/ARNI use for CTRCD, *n*/N (%)	114/179 (63.7)
Beta-blocker use for CTRCD, *n*/N (%)	129/179 (72.1)
Mineralocorticoid receptor antagonist use for CTRCD, *n*/N (%)	30/179 (16.8)
SGLT2 inhibitor use for CTRCD, *n*/N (%)	5/179 (2.8)
Diuretic therapy for congestion/HF, *n*/N (%)	40/179 (22.3)
LVEF recovery after CTRCD	
Complete LVEF recovery, *n*/N (%)	124/179 (69.3)
Partial LVEF recovery, *n*/N (%)	30/179 (16.8)
No documented LVEF recovery, *n*/N (%)	15/179 (8.4)
Recovery status unavailable, *n*/N (%)	10/179 (5.6)
Estimated time to any LVEF recovery, months *	4 (2–8)
Estimated time to complete LVEF recovery, months *	4 (2–7)
Estimated time to partial LVEF recovery, months *	6 (3–10)

Data are presented as *n*/N (%) or median [IQR], as appropriate. Percentages for CTRCD management and LVEF recovery variables were calculated among the 179 patients who developed CTRCD. Treatment categories are not mutually exclusive because more than one cardioprotective/HF therapy could be initiated in the same patient. LVEF recovery categories are mutually exclusive and collectively account for all patients who developed CTRCD, including those for whom recovery status was unavailable. * Time-to-recovery estimates were calculated among patients with available temporal information documenting subsequent LVEF recovery. ACE-i, angiotensin-converting enzyme inhibitor; ARB, angiotensin receptor blocker; ARNI, angiotensin receptor–neprilysin inhibitor; CTRCD, cancer-therapy-related cardiac dysfunction; HF, heart failure; IQR, interquartile range; LVEF, left ventricular ejection fraction; SGLT2, sodium–glucose cotransporter 2.

**Table 8:** Oncological outcomes at the latest available follow-up in the 10-year cohort.

Oncological Outcome	10-Year Cohort (*n* = 850)
No evidence of disease/disease-free follow-up, *n* (%)	437 (51.4)
Metastatic disease with complete/partial response or stable disease, *n* (%)	164 (19.3)
Metastatic disease with documented progression/palliative course, *n* (%)	124 (14.6)
Ongoing neoadjuvant/adjuvant curative-intent treatment, *n* (%)	75 (8.8)
Other/insufficiently classifiable oncological outcome, *n* (%)	50 (5.9)
Pathological complete response after neoadjuvant therapy, *n* (%) *	70 (8.2)

Data are presented as *n* (%). Oncological outcome categories refer to the latest available clinical assessment and are mutually exclusive. * Pathological complete response after neoadjuvant therapy represents an additional treatment-response variable and is not mutually exclusive with the main oncological outcome categories; therefore, it is not included in their cumulative percentage. n, number of patients.

**Table 9:** Univariable logistic regression analysis of clinical, oncological, treatment-related, and baseline echocardiographic factors associated with CTRCD.

Variable	OR	95% CI	*p*-Value
**Clinical variables**			
Age, per 10 years	1.12	0.88–1.43	0.360
BMI, per 5 kg/m^2^	1.58	0.93–2.70	0.090
Hypertension	1.28	0.86–1.91	0.225
Diabetes mellitus	1.34	0.68–2.64	0.398
Dyslipidemia	1.18	0.79–1.76	0.418
Current/former smoking	1.32	0.54–3.25	0.544
Charlson Comorbidity Index, per 1 point	1.32	1.10–1.58	**0.003**
**Oncological/treatment variables**			
Tumor grade, per 1 grade increase	1.16	0.52–2.60	0.717
Anthracycline pretreatment (yes vs. no)	1.13	0.45–2.85	0.790
AC cycles, per cycle	1.00	0.80–1.24	0.977
Total anti-HER2 duration, per 5 cycles	1.37	1.09–1.71	**0.006**
**Baseline echocardiographic variables**			
Baseline LVEF, per 1% decrease	0.97	0.89–1.05	0.449
Baseline LV-GLS magnitude, per 1% decrease	0.67	0.39–1.15	0.145

Odds ratios and corresponding 95% CIs were derived from univariable binary logistic regression analyses with CTRCD as the dependent variable. Continuous variables were modeled according to the increments specified in the table. Statistically significant *p*-values are shown in bold. AC, anthracycline-containing chemotherapy; BMI, body mass index; CI, confidence interval; CTRCD, cancer-therapy-related cardiac dysfunction; HER2, human epidermal growth factor receptor 2; LV-GLS, left ventricular global longitudinal strain; LVEF, left ventricular ejection fraction; OR, odds ratio.

**Table 10:** Multivariable logistic regression analysis of factors associated with cancer-therapy-related cardiac dysfunction after mutual adjustment.

Variable	Adjusted OR	95% CI	*p*-Value
Charlson Comorbidity Index, per 1 point	1.35	1.12–1.63	**0.002**
Total anti-HER2 duration, per 5 cycles	1.41	1.10–1.80	**0.006**

Adjusted odds ratios and corresponding 95% CIs were derived from a parsimonious multivariable binary logistic regression model with CTRCD as the dependent variable. CCI was modeled per one-point increase, whereas cumulative anti-HER2 exposure was modeled per five additional treatment cycles. Statistically significant *p*-values are reported in bold. CCI, Charlson Comorbidity Index; CI, confidence interval; CTRCD, cancer-therapy-related cardiac dysfunction; HER2, human epidermal growth factor receptor 2; OR, odds ratio.

## Data Availability

The dataset generated and analyzed during the present study will be made openly accessible through the Zenodo repository (https://zenodo.org).

## Supplementary Materials

The following supporting information can be downloaded at: https://www.mdpi.com/article/10.3390/jcm15197700/s1, File S1: Full study protocol [25,26]; File S2: Approval document issued by the Comitato Etico Territoriale Lombardia 5 (CET Lombardia 5).
