# Supplementary material for: Comorbidity Burden and Cumulative Anti-HER2 Exposure Associated with Cardiac Dysfunction in Breast Cancer: A Real-World Cardio-Oncology Cohort
Source: J Clin Med. 2026 Oct 5;15(19):7700. doi: 10.3390/jcm15197700 (PMC13665819; doi:10.3390/jcm15197700)
Supplement: Supplementary file 1 [file jcm-15-07700-s001.zip › Supplementary Material File S1.pdf]

Cardiotoxicity in cancer patients treated with modern anticancer therapies: a retro-prospective monocentric study.

ACRONYME: Project "CARTOX-PREDICT"

**Authors:**	*Andrea Sonaglioni (P.I.), Division of Cardiology, MultiMedica*<br>*Emanuela Fossile (CoP.I.), Division of Oncology, MultiMedica*
**Program:**	*Cardio-Oncology Research*
**Institutions:**	*MultiMedica*
**Corresponding Author:**	*Andrea Sonaglioni M.D.*<br>*Division of Cardiology*<br>*MultiMedica – Ospedale San Giuseppe*<br>*andrea.sonaglioni@multimedica.it*

Protocol/version #:	*1.1*
Current Version Date:	*07/10/2025*
Previous IEC Approved Version Dates:	

CONFIDENTIAL

This document is confidential and the property of individual. No part of it may be transmitted, reproduced, published, or used by other persons without prior written authorization from the author.

List of Abbreviations

2D, two-dimensional; BMI, body mass index; BSA, body surface area; CKD, chronic kidney disease; COPD, chronic obstructive pulmonary disease; CRP, C-reactive protein; CTRCD, cancer therapy-related cardiac dysfunction;; eGFR, estimated glomerular filtration rate; GLS, global longitudinal strain; HDL, high-density lipoprotein; HER2, human epidermal growth factor receptor 2; HF, heart failure; HS, high-sensitivity; ICC, intraclass correlation coefficient; ICIs, immune checkpoint inhibitors; IDA, initial data analysis; IRAEs, immune-related adverse events; IVC, inferior vena cava; LAVi, left atrial volume index; LDL, low-density lipoprotein; LV, left ventricular; LVEDVi, left ventricular end-diastolic volume index; LVEF, left ventricular ejection fraction; LVESVi, left ventricular end-systolic volume index; LVFP, left ventricular filling pressure; LVMi, left ventricular mass index; NT-proBNP, N-terminal pro-B-type natriuretic peptide; OSAS, obstructive sleep apnea syndrome; RV, right ventricular; RVIT, right ventricular inflow tract; RWT, relative wall thickness; sPAP, systolic pulmonary artery pressure; TAPSE, tricuspid annular plane systolic excursion; TSH, thyroid-stimulating hormone; TTE, transthoracic echocardiography; VEGF, vascular endothelial growth factor,

Table of Contents

S1 INTRODUCTION/SIGNIFICANCE 4,5

S1.1 Anthracycline-related cardiotoxicity........ 4
S1.2 Anti-HER2-related cardiotoxicity........ 4
S1.3 Fluoropyrimidines induced-cardiotoxicity........ 4
S1.4 VEGF inhibitors-induced cardiotoxicity........ 5
S1.5 ICI-related cardiotoxicity........ 5
S1.6 Rationale........ 5

S2 STUDY OBJECTIVES........ 6

S2.1 PRIMARY OBJECTIVE 6
S2.2 SECONDARY OBJECTIVE 6

S3 PATIENTS AND METHODS 6-12

S3.1 STUDY DESIGN 6
S3.1.1 General Design 6
S3.1.2 Primary Outcome Variable........ 6
S3.1.3 Secondary Outcome Variables........ 6
S3.2 SUBJECT SELECTION AND WITHDRAWAL 6
S3.2.1 Inclusion Criteria 7
S3.2.2 Exclusion Criteria 7
S3.3 STUDY PROCEDURES 8
S3.3.1 *Clinical and laboratory data* 8
S3.3.2 *Resting transthoracic echocardiography*........ 8
S3.3.3 *Speckle tracking echocardiography*........ 9
Schedule of Study Procedures........ 10
S3.4 Statistical Plan........ 11
S3.4.1 Sample Size Determination........ 11
S3.4.2 Statistical Methods........ 12

S4 DATA HANDLING AND RECORD KEEPING........ 12

S4.1 CONFIDENTIALITY 12
S4.2 RECORDS RETENTION 12

S5**PUBLICATION PLAN 13**

S6 REFERENCES........ 14-20

S1 Introduction

This document is a clinical research protocol and the described study will be conducted in accordance with the ethical standards of our Institutional Research Committee and with the 1964 Helsinki Declaration and its later amendments or comparable ethical standards.

Recent innovations in anticancer therapy have led to exciting progress in prolonged patient survival. However, these survival advances have also highlighted the adverse effects that may occur with anticancer therapy, one of the most common being cardiotoxicity [1].

During the last few years, several studies have been performed to determine the incidence of cancer therapy-related cardiac dysfunction (CTRCD) and to identify the independent predictors of CTRCD related to anthracyclines, anti-human epidermal growth factor receptor 2 (HER2), fluoropyrimidines, vascular endothelial growth factor (VEGF) inhibitors and/or immune checkpoint inhibitors (ICIs).

S1.1 Anthracycline-related cardiotoxicity

Anthracycline-related cardiotoxicity may be categorized in early onset cardiotoxicity and late onset cardiotoxicity. The former represents 98% of cases, occurs within the first year after treatment and is generally associated with an asymptomatic decline in left ventricular ejection fraction (LVEF); the latter may appear in patients exposed to more than 1 year of treatment and is characterized by hypokinetic and/or dilated cardiomyopathy with overt heart failure (HF) symptoms [2,3]. A 2013 meta-analysis of 22,815 patients treated with anthracyclines reported clinically overt cardiotoxicity and subclinical cardiotoxicity in 6 and 18% of cases, respectively [4]. In a recent review, cardiotoxicity was detected in 9% of patients who underwent anthracycline chemotherapy, and the great majority of cases (98% of total) was reported within the first year after the completion of chemotherapy [5]. Pre-existing cardiovascular risk factors, particularly hypertension, diabetes mellitus, or obesity, have been associated with increased incidences of anthracycline-induced cardiotoxicity [6]. The principal independent predictors of anthracycline-related cardiotoxicity are: cumulative anthracycline dose, chest radiotherapy, African-American ethnicity, very young or very old age, diabetes, hypertension, very high or very low body weight, or severe comorbidities [4].

S1.2 Anti-HER2-related cardiotoxicity

The overall incidence of trastuzumab-related cardiotoxicity has been reported to range between 8 and 35.6% in various studies [7,8]. The most common event observed in patients receiving trastuzumab treatment is an asymptomatic or mildly symptomatic left ventricular (LV) systolic function decline [9].

The main risk factors for trastuzumab-induced cardiotoxicity are: older age [10], previous or concurrent anthracycline use [8], length of trastuzumab treatment, obesity [11], African-American ethnicity [12], diabetes mellitus [13], dyslipidemia [13], decreased glomerular filtration rate [14], pre-existing cardiac dysfunction with a low baseline LVEF [7], use of antihypertensives [9] and in patients with higher HFA-ICOS score [15]. For these reasons, cardiovascular mortality observed in breast cancer patients is higher than in the general population [16]. Trastuzumab-related cardiotoxicity has been reported to be reversible in most cases, particularly when trastuzumab administration is discontinued and/or cardioprotective treatment is initiated [17].

S1.3 Fluoropyrimidines induced-cardiotoxicity

Fluoropyrimidines may induce cardiotoxicity, with an incidence ranging from 1% to 18%. The most common symptom of fluoropyrimidine cardiotoxicity is chest pain [18]. Less common

manifestations include myocardial infarction, reversible cardiomyopathy, myopericarditis, congestive heart failure, tachyarrhythmias, coronary dissection, cardiogenic shock, silent cardiac ischemia, and sudden death [20]. The main recognized risk factors for fluoropyrimidine cardiotoxicity are older age, concurrent radiotherapy or history of chest radiotherapy, concurrent administration of cardiotoxic drugs, history of cardiovascular diseases or the presence of cardiovascular risk factors such as hypertension, dyslipidemia, diabetes mellitus, and smoking [21]. Studies have shown that the continuous infusion of 5-fluorouracil compared with bolus administration is associated with a higher incidence for cardiac adverse event [22].

S1.4 VEGF inhibitors-induced cardiotoxicity

VEGF inhibitors-induced myocardial toxicity ranges from asymptomatic forms to irreversible heart failure, cardiogenic shock and death [2,23]. The incidence of cardiac dysfunction varies between 3 and 15% and heart failure may occur in 10% of the patients [24-26]. The risk for cardiotoxicity is higher in patients with preexisting hypertension, diabetes, coronary artery disease, atherosclerosis and previous chemotherapy or radiotherapy [2,24,27].

Approximately 180 cancer patients per year are referred to the Division of Oncology of our Institution. All the principal anti-cancer treatments are administered to these patients.

S1.5 ICI-related cardiotoxicity

ICI may induce several immune-related adverse events (IRAEs), which may also affect the cardiovascular system [28-30]. ICI-related myocarditis is undoubtedly the most severe type of IRAEs, usually occurring within the first 3 months of therapy, with an incidence of 0.09–2.4% [31-33] and a mortality rate of 25–50% [34-36]. Recent echocardiographic studies have demonstrated an early attenuation of conventional echocardiographic parameters and innovative myocardial strain indices involving both ventricles after ICI treatment [37-40]. ICI combination therapy (at least one anti-PD-1/PD-L1 with anti-CTLA-4) [41,42] have been associated with increased risk of developing cardiovascular IRAEs. Cardiovascular irAEs are frequently reported among patients with melanoma or lung cancer [43]. Other risk factors for IRAEs are age <60 years, high body mass index, women on CTLA4 and men on PD-1/PD-L1 agents, and chronic smokers. Patients with significant kidney (Stage IV-V), cardiac (heart failure, coronary artery disease, myocardial infarction, hypertension), and lung (asthma, pulmonary fibrosis, and chronic obstructive pulmonary disease) are at a higher risk of respective organ-specific irAEs. High neutrophil-lymphocyte ratio, eosinophil/monocyte/lymphocyte counts; TSH and troponins at diagnosis and drop in the white count and lymphocyte count can predict irAE [44].

S1.6 Rationale

To date, the incidence and independent predictors of CTRCD related to anthracyclines, anti-HER2, fluoropyrimidines, VEGF inhibitors and/or ICIs have been assessed by randomized controlled trials or observational community practice-based studies, with great variability and wide ranges for each anti-cancer treatment [7,45]. Conversely, the real incidence of CTRCD related to anti-cancer treatments is not known. Accordingly, the present study has been primarily designed to investigate the incidence of cardiotoxicity related to anthracyclines or anti-HER2-therapies or fluoropyrimidines or VEGF inhibitors and/or ICIs in a large retrospective monocentric cohort of cancer patients, followed at IRCCS MultiMedica, Milan, Italy, over a ten-year timeframe. Secondly, we aim at identifying the specific echocardiographic pattern of CTRCD assessed by conventional transthoracic echocardiography (TTE) implemented with speckle tracking echocardiography (STE) analysis of both ventricles and atria in a prospective cohort of cancer

patients treated with anti-HER2-therapies alone or combined with anthracyclines or ICIs selected from the same Center.

S2 Study Objectives

S2.1 Primary Objective

To estimate the incidence of cardiotoxicity related to anthracyclines or anti-HER2-therapies or fluoropyrimidines or VEGF inhibitors and/or ICIs in a large retrospective monocentric cohort of cancer patients, followed at IRCCS MultiMedica, Ospedale San Giuseppe, Milan, Italy, over a ten-year timeframe.

S2.2 Secondary Objective

To identify the specific echocardiographic pattern of biventricular and biatrial CTRCD in a prospective cohort of cancer patients treated with anti-HER2-therapies alone or combined with anthracyclines or ICIs, selected from the same Center.

S3 Patients and Methods

S3.1 Study Design

S3.1.1 General Design

Observational, retro-prospective, monocentric study.

S3.1.2 Primary Outcome Variable

The present study primarily aims at estimating the rates of cardiotoxicity related to anthracyclines or anti-HER2-therapies or fluoropyrimidines or VEGF inhibitors and/or ICIs in a large retrospective monocentric cohort of cancer patients, followed at IRCCS MultiMedica, Milan, Italy, over a ten-year timeframe.

S3.1.3 Secondary outcome Variable

The present study also aims at identifying the specific TTE and STE-derived pattern of biventricular and biatrial CTRCD in a prospective cohort of cancer patients treated with anti-HER2-therapies alone or combined with anthracyclines or ICIs, , selected from the same Center.

S3.2 Subject Selection and Withdrawal

S3.2.1 Inclusion Criteria for the retrospective study

All adult cancer patients who underwent treatment with anthracyclines or anti-HER2 or fluoropyrimidines or VEGF inhibitors and/or ICIs, at the Division of Oncology of the IRCCS MultiMedica between the 1st January 2014 and the 31st December 2024 inclusive.

Following cohorts of cancer patients will be retrospectively analyzed:

1) patients affected by breast cancer who were treated with anthracyclines (4 cycles every 3 weeks in the neoadjuvant and adjuvant phases, 6 cycles in the metastatic phase) and/or anti-HER2 (every 3 weeks for 12 months in the adjuvant phase, until disease progression or unacceptable toxicity in the metastatic phase) and/or ICIs (4 cycles every 3 weeks in the neoadjuvant phase, 9 cycles every 3 weeks in the adjuvant phase, until disease progression or unacceptable toxicity in the metastatic setting);
2) patients affected by colorectal cancer who received treatment with fluoropyrimidines (12 cycles every 2 weeks in the adjuvant phase) or VEGF inhibitors (until disease progression or unacceptable toxicity in the metastatic phase) and/or ICIs (in the metastatic phase for two years);
3) patients affected by lung cancer who were treated with ICIs in the neoadjuvant phase (4 cycles every 3 weeks), in the adjuvant phase (every 3 weeks for 2 years) and in the metastatic phase (until disease progression or unacceptable toxicity) and/or with VEGF inhibitors in the adjuvant phase (every 3 weeks for 3 years) and in the metastatic phase (until disease progression or unacceptable toxicity);
4) patients affected by renal cancer treated with ICIs in the metastatic phase until disease progression or unacceptable toxicity;
5) patient affected by melanoma treated with ICIs in the adjuvant phase for 1 year and in the metastatic phase until disease progression or unacceptable toxicity.

S3.2.2 Exclusion Criteria for the retrospective study

Age ≤18 yrs; cancer patients referred to the Division of the IRCCS MultiMedica before the 1st January 2014 or after the 31st December 2024; incomplete clinical and/or echocardiographic data.

S3.2.3 Inclusion Criteria for the prospective study

Cancer patients candidates to treatment with anti-HER2-therapies alone or combined with anthracyclines or ICIs, followed-up at the Division of Oncology of the IRCCS MultiMedica, evaluated by TTE implemented with biventricular and biatrial STE analysis.

S3.2.4 Exclusion Criteria for the prospective study

Age ≤18 yrs; hemodynamic instability; poor echocardiographic acoustic windows; patient’s refusal to participate in the present clinical research.

All cancer patients will be selected from the Division of Oncology of the IRCCS MultiMedica.

S3.3 Study Procedures

For the retrospective study, all clinical, laboratory and echocardiographic data concerning the baseline evaluation (before initiating anti-cancer treatment) and the follow-up visits (at 1 month, 3 months and 6 months from the beginning of the treatment) will be collected by accessing medical records available in the hospital archive.

For the prospective study, each cancer patient will undergo a basal evaluation (T0, before starting treatment) and three follow-up visits: at one month (T1), three months (T2), and 6 months (T3) during treatment, respectively. At each time point, cancer patients will undergo an oncology visit (as per routine clinical practice and standard of care at our Center), blood sampling (as per routine clinical practice and standard of care at our Center), and conventional TTE (as per routine clinical practice and standard of care at our Center) implemented with STE analysis of both ventricles and atria (procedure in addition to normal clinical practice). The study duration is estimated to be in 12 months

S3.3.1 Clinical and laboratory data

For both the retrospective and prospective study, the following data will be collected from patients' hospital medical charts: age; gender; anthropometrics [body surface area (BSA) and body mass index (BMI)]; prevalence of relevant cardiovascular risk factors (hypertension, smoking, type 2 diabetes and dyslipidemia); type of cancer, cancer stage and the specific cancer treatment; main comorbidities [chronic kidney disease (CKD), defined as an estimated glomerular filtration rate (eGFR) <60 ml/min/m) [46], history of coronary artery disease (previous acute coronary syndrome, previous percutaneous and/or surgical coronary revascularization), peripheral arteriopathy, previous stroke and/or transient ischemic attack, cognitive impairment, chronic obstructive pulmonary disease (COPD), obstructive sleep apnea syndrome (OSAS), hypothyroidism, anemia (defined as hemoglobin <12 g/dl for females or 13 g/dl for males)]; blood tests comprehensive of complete blood count, serum creatinine and eGFR, serum levels of glucose, sodium, potassium, uric acid, calcium, total bilirubin, total cholesterol, high-density lipoprotein (HDL) cholesterol, low-density lipoprotein (LDL) cholesterol, thyroid-stimulating hormone (TSH), C-reactive protein (CRP), N-terminal pro-B-type natriuretic peptide (NT-proBNP), high-sensitivity (HS) troponine; blood pressure measurements; current medical treatment.

S3.3.2 Resting transthoracic echocardiography

For both the retrospective and prospective study, all echocardiograms are performed with the participant in left-lateral decubitus, and parameters measured according to the Recommendations of the American Society of Echocardiography and the European Association of Cardiovascular Imaging [47,48]. Echocardiographic measurements are indexed to BSA.

Following conventional two-dimensional (2D) echoDoppler parameters are recorded at basal evaluation and during follow-up period: the aortic root and ascending aorta diameters by using the "leading edge-to-leading edge" convention; the relative wall thickness (RWT), calculated with the formula RWT = 2 posterior wall thickness/LV internal diameter at end-diastole; the left ventricular mass index (LVMi), calculated by the Devereux's formula; the left ventricular end-diastolic volume index (LVEDVi) and the left ventricular end-systolic

volume indexed (LVESVi); the left ventricular ejection fraction (LVEF), estimated with the biplane modified Simpson's method, as index of LV systolic function [47]; the left atrial volume index (LAVi); the right ventricular inflow tract (RVIT); the tricuspid annular plane systolic excursion (TAPSE), as index of right ventricular (RV) systolic function; finally, the inferior vena cava (IVC) diameter during normal respiration.

Doppler measurements include the E/A ratio and the E/average e' ratio, as indices of LV diastolic function and left ventricular filling pressure (LVFP), respectively [48]. Systolic pulmonary artery pressure (sPAP) is derived by the modified Bernoulli equation, where sPAP = 4 × (tricuspid regurgitation velocity)^2 + right atrial pressure [49]. The latter is estimated from IVC diameter and collapsibility.

Degree of concomitant valvulopathy is assessed according to the AHA/ACC recommendations for the assessment of valvular regurgitation [50].

S3.3.3 Speckle-tracking echocardiography

For both the retrospective and prospective study, immediately after conventional transthoracic echocardiography (TTE), high-resolution speckle-tracking strain analyses are performed on 2D images in the following order: apical four-chamber, two-chamber and three-chamber views for LV longitudinal strain, using the automated function imaging and the Q-Analysis module [51]. According to Philips QLAB software, the LV wall is divided into seven segments in each apical view. Left ventricular peak systolic strain is defined as the point of maximal systolic shortening for longitudinal and circumferential strain. A single bull's-eye summary for LV global longitudinal strain (GLS) and LV global circumferential strain (GCS) is obtained, presenting the analysis for each segment along with the assessment of global LV strain. Early peak diastolic strain rate is obtained from longitudinal and circumferential measurements [51].

RV-GLS is measured from the average of individual strain curves obtained from the apical four-chamber view. Right ventricular free wall longitudinal strain is calculated as the mean of RV lateral basal, mid and apical segments, with exclusion of septal segments [52].

Left-atrial (LA) strain is calculated by using a "biplane method". The following measurements are performed: the peak positive LA longitudinal strain corresponding to left-atrial conduit strain (LAScd), the peak negative LA longitudinal strain corresponding to left-atrial contractile strain (LASct) and the sum of the two peaks corresponding to left-atrial reservoir strain (LASr). From the atrial strain measurements, the strain rate curves are derived [53].

Right-atrial reservoir strain (RASr) is assessed from the apical four-chamber view by placing the markers on the edges of the tricuspid annulus and to the right-atrial roof.

Absolute values superior to 20% for LV-GLS [54], 23.3% for LV-GCS [55], 20% for RV-GLS [56], 39% for LASr [57] and 35% for RASr [58] are considered as the cut-off values of normality for comparison.

Table S1 and S2 *summarize all the visits and procedures, detailed with timing, planned for each individual included in the retrospective and prospective study, respectively.*

Table S1: Schedule of Study Procedures for the retrospective study

**Study Phase**	**Screening**	**Follow-up (starting from anti-cancer treatment)**		
**Visit number**	**Basal Evaluation anti-cancer treatment 01/01/2014-01/01/2024**	**Time 1 (1 month**	**Time 2 (3 months)**	**Time 3 (6 months)**
Anthropometrics: BSA, BMI^	X	X	X	X
Blood tests^<br>serum creatinine and eGFR, serum levels of glucose, sodium, potassium, uric acid, calcium, total bilirubin, total cholesterol, high-density lipoprotein (HDL) cholesterol, low-density lipoprotein (LDL) cholesterol, thyroid-stimulating hormone (TSH), C-reactive protein (CRP), N-terminal pro-B-type natriuretic peptide (NT-proBNP), high-sensitivity (HS) troponine	X	X	X	X
Conventional TTE^	X	X	X	X
STE*	X	X	X	X

^*Normal clinical practice; *Procedure in addition to normal clinical practice*

Table S2: Schedule of Study Procedures for the prospective study

**Study Phase**	**Screening**	**Follow-up (starting from anti-cancer treatment)**		
**Visit number**	**Basal Evaluation**	**Time 1 (1 month**	**Time 2 (3 months)**	**Time 3 (6 months)**
Review Inclusion/Exclusion Criteria	X			
Informed Consent	X			
Oncology visit^	X	X	X	X
Demographics/Medical History^	X	X	X	X
Anthropometrics: BSA, BMI^	X			
Physical Examination^	X	X	X	X
Vital Signs: BP, HR^	X	X	X	X

Blood tests^<br>serum creatinine and eGFR, serum levels of glucose, sodium, potassium, uric acid, calcium, total bilirubin, total cholesterol, high-density lipoprotein (HDL) cholesterol, low-density lipoprotein (LDL) cholesterol, thyroid-stimulating hormone (TSH), C-reactive protein (CRP), N-terminal pro-B-type natriuretic peptide (NT-proBNP), high-sensitivity (HS) troponine	X	X	X	X
Conventional TTE^	X	X	X	X
STE*	X	X	X	X

*^Normal clinical practice; *Procedure in addition to normal clinical practice*

S3.4 Statistical Plan

S3.4.1 Sample Size Determination for the retrospective study

From our preliminary data, CTCRD was detected in about 20% of cancer patients. Considering that approximately 1800 patients have been followed-up at the Division of Oncology of the IRCCS MultiMedica between the 1st January 2014 and the 31st December 2024, such sample size will reach about 90% of power in detect a difference of ±3%, using a two-sided, one-sample exact test, with a Type I error rate (α) of 5%.

S3.4.2 Sample Size Determination for the prospective study

Based on our preliminary observation, the effect size (f = 0.25) has been calculated as the difference between the hypothesized means of LV-GLS at time 0 (20%) vs time 3 (19.5%) divided by the pooled standard deviations (SD) of LV-GLS measurements at time 0 and time 3 (pooled SD = 2). Running a power analysis on a repeated measures ANOVA with three measurements, a power of 0.80, an alpha level of 0.05, and a medium effect size (f= 0.25), the required sample size is 28 patients.

S3.4.3 Statistical Methods

Initial data analysis (IDA) will be performed to ensure that the later statistical analysis can be performed efficiently and to minimize the risk of incorrect or misleading results [59]. IDA can be divided into 3 main steps: 1. Data cleaning is the identification of inconsistencies, and missing in data and the resolution of any such issues. 2. Data screening is the description of the data properties. 3. Documentation and reporting preserve the information for later statistical analysis and models. The normality of the distribution of continuous variables will be tested using the Kolmogorov–Smirnov test and by visual inspection of qq-plot. Normally distributed variables will be summarized as mean ± standard deviation, while not normally distributed data will be expressed as median with quartiles (first - third). Categorical data will be summarized by frequencies and percentages and compared between groups by Chi Square test or Fisher's exact test, as appropriate. Correlations between continuous variables will be evaluated according to Pearson R or Spearman Rho, depending on the distribution.

CTRCD will be defined according to the European Guidelines [2] and will be classified in mild, moderate, and severe, depending on the degree of LVEF and/or LV-GLS impairment. Mild CTRCD is defined as LVEF ≥50% and new relative decline in LV-GLS by >15% from baseline and/or new rise in cardiac biomarkers (troponin and natriuretic peptides). Moderate CTRCD is defined as new LVEF reduction by ≥10 percentage points to an LVEF of 40–49% or new LVEF reduction by <10 percentage points to an LVEF of 40–49% and either new relative decline in LV-GLS by >15% from baseline or new rise in cardiac biomarkers. Severe CTRCD is defined as new LVEF reduction to <40%. The prevalence of CTRCD detected in the retrospective cohort of cancer patients included will be expressed as crude numbers, and percentages.

Univariate and multivariate logistic regression analyses will be performed to identify the independent predictors of CTRCD at the end of the follow-up period. For each variable investigated, correspondent odds ratios with 95% confidence intervals (CIs) will be calculated. Only the variables with a p-value<0.10 on univariate analyses will be included in the multivariate logistic regression model.

Test-retest reliability for repeated measurements will be performed to assess the consistency and reproducibility of LVEF and LV-GLS parameters measured in a limited subgroup of 30 cancer patients who underwent trastuzumab treatment. Test-retest reliability will be assessed for three repeated measurements [at time 0 (baseline), time 1 (one month) and time 2 (three months) from the beginning of the treatment] by calculating the correlation coefficient between the scores of the three assessments. The reliability will be interpreted using an intraclass correlation coefficient (ICC). An ICC below 0.5 indicates poor reliability, between 0.5 and 0.75 moderate reliability, between 0.75 and 0.9 good reliability, and any value above 0.9 indicated excellent reliability [60].

Statistical analysis will be performed with SPSS version 28 (SPSS Inc., Chicago, Illinois, USA), with two-tailed p values below 0.05 deemed statistically significant.

S4 Data Handling and Record Keeping

Data to be collected**:** demographic, anthropometric, clinical, biochemical and echocardiographic data (as already listed in the Study Procedures section). All electronic data will be collected from the Hospital repository and will be reported into an excel worksheet, at baseline and during each follow-up visit. The principal investigator will collect all the data.

Data Controller will publish on its website the information notice on the processing of personal data for the Study and the related data protection impact assessment, notifying the Data Protection Authority, as provided for by the aforementioned Code of Ethics for processing for statistical or scientific research purposes.

S4.1 Confidentiality

All patient data will be analyzed in anonymous form and will be accessible only to the Principal Investigator, that is obliged to keep the professional secret.

S4.2 Records Retention

All medical records are available in the repository of the San Giuseppe MultiMedica IRCCS Hospital.

Moreover, data extracted from the present study will be publicly available on Zenodo after the publication (https://zenodo.org).

S5 Publication Plan

We intend to present preliminary results of the present study within the next six months

Only aggregate data without individually identifiable information will be published.

We intend to publish the present study within the next 12 months.

S6 References

1) Conway A, McCarthy AL, Lawrence P, Clark RA. The prevention, detection and management of cancer treatment-induced cardiotoxicity: a meta-review. BMC Cancer. 2015 May 7;15:366. doi: 10.1186/s12885-015-1407-6. PMID: 25948399; PMCID: PMC4427936.
2) Lyon AR, López-Fernández T, Couch LS, Asteggiano R, Aznar MC, Bergler-Klein J, Boriani G, Cardinale D, Cordoba R, Cosyns B, Cutter DJ, de Azambuja E, de Boer RA, Dent SF, Farmakis D, Gevaert SA, Gorog DA, Herrmann J, Lenihan D, Moslehi J, Moura B, Salinger SS, Stephens R, Suter TM, Szmit S, Tamargo J, Thavendiranathan P, Tocchetti CG, van der Meer P, van der Pal HJH; ESC Scientific Document Group. 2022 ESC Guidelines on cardio-oncology developed in collaboration with the European Hematology Association (EHA), the European Society for Therapeutic Radiology and Oncology (ESTRO) and the International Cardio-Oncology Society (IC-OS). Eur Heart J. 2022 Nov 1;43(41):4229-4361. doi: 10.1093/eurheartj/ehac244. Erratum in: Eur Heart J. 2023 May 7;44(18):1621. doi: 10.1093/eurheartj/ehad196. PMID: 36017568.
3) Cardinale D, Colombo A, Bacchiani G, Tedeschi I, Meroni CA, Veglia F, Civelli M, Lamantia G, Colombo N, Curigliano G, Fiorentini C, Cipolla CM. Early detection of anthracycline cardiotoxicity and improvement with heart failure therapy. Circulation. 2015 Jun 2;131(22):1981-8. doi: 10.1161/CIRCULATIONAHA.114.013777. Epub 2015 May 6. PMID: 25948538.
4) Lotrionte M, Biondi-Zoccai G, Abbate A, Lanzetta G, D'Ascenzo F, Malavasi V, Peruzzi M, Frati G, Palazzoni G. Review and meta-analysis of incidence and clinical predictors of anthracycline cardiotoxicity. Am J Cardiol. 2013 Dec 15;112(12):1980-4. doi: 10.1016/j.amjcard.2013.08.026. Epub 2013 Sep 25. PMID: 24075281.
5) Sawicki KT, Sala V, Prever L, Hirsch E, Ardehali H, Ghigo A. Preventing and Treating Anthracycline Cardiotoxicity: New Insights. Annu Rev Pharmacol Toxicol. 2021 Jan 6;61:309-332. doi: 10.1146/annurev-pharmtox-030620-104842. PMID: 33022184.
6) Cai F, Luis MAF, Lin X, Wang M, Cai L, Cen C, Biskup E. Anthracycline-induced cardiotoxicity in the chemotherapy treatment of breast cancer: Preventive strategies and treatment. Mol Clin Oncol. 2019 Jul;11(1):15-23. doi: 10.3892/mco.2019.1854. Epub 2019 May 8. PMID: 31289672; PMCID: PMC6535635.
7) Onitilo AA, Engel JM, Stankowski RV. Cardiovascular toxicity associated with adjuvant trastuzumab therapy: prevalence, patient characteristics, and risk factors. Ther Adv Drug Saf. 2014 Aug;5(4):154-66. doi: 10.1177/2042098614529603. PMID: 25083270; PMCID: PMC4110857.
8) Henry ML, Niu J, Zhang N, Giordano SH, Chavez-MacGregor M. Cardiotoxicity and Cardiac Monitoring Among Chemotherapy-Treated Breast Cancer Patients. JACC Cardiovasc Imaging. 2018 Aug;11(8):1084-1093. doi: 10.1016/j.jcmg.2018.06.005. PMID: 30092967; PMCID: PMC6149535.
9) de Azambuja E, Ponde N, Procter M, Rastogi P, Cecchini RS, Lambertini M, Ballman K, Aspitia AM, Zardavas D, Roca L, Gelber RD, Piccart-Gebhart M, Suter T. A pooled analysis of the cardiac events in the trastuzumab adjuvant trials. Breast Cancer Res Treat. 2020 Jan;179(1):161-171. doi: 10.1007/s10549-019-05453-z. Epub 2019 Oct 11. PMID: 31605311; PMCID: PMC6989393.

10) Chavez-MacGregor M, Zhang N, Buchholz TA, Zhang Y, Niu J, Elting L, Smith BD, Hortobagyi GN, Giordano SH. Trastuzumab-related cardiotoxicity among older patients with breast cancer. J Clin Oncol. 2013 Nov 20;31(33):4222-8. doi: 10.1200/JCO.2013.48.7884. Epub 2013 Oct 14. PMID: 24127446; PMCID: PMC3821011.
11) Toukabri I, Ben Said A, Hamdi A, Aloulou A, Ben Ayed W, Cherif I, Limayem I. Prevalence and risk factors of trastuzumab induced cardiotoxicity in Tunisian HER2-positive breast cancer patients. J Oncol Pharm Pract. 2023 Apr;29(3):613-618. doi: 10.1177/10781552221076407. Epub 2022 Jan 28. PMID: 35088630.
12) Al-Sadawi M, Hussain Y, Copeland-Halperin RS, Tobin JN, Moskowitz CS, Dang CT, Liu JE, Steingart RM, Johnson MN, Yu AF. Racial and Socioeconomic Disparities in Cardiotoxicity Among Women With HER2-Positive Breast Cancer. Am J Cardiol. 2021 May 15;147:116-121. doi: 10.1016/j.amjcard.2021.02.013. Epub 2021 Feb 20. PMID: 33617819; PMCID: PMC8076074.
13) Kosalka P, Johnson C, Turek M, Sulpher J, Law A, Botros J, Dent S, Aseyev O. Effect of obesity, dyslipidemia, and diabetes on trastuzumab-related cardiotoxicity in breast cancer. Curr Oncol. 2019 Jun;26(3):e314-e321. doi: 10.3747/co.26.4823. Epub 2019 Jun 1. PMID: 31285674; PMCID: PMC6588065.
14) Russo G, Cioffi G, Di Lenarda A, Tuccia F, Bovelli D, Di Tano G, Alunni G, Gori S, Faggiano P, Tarantini L. Role of renal function on the development of cardiotoxicity associated with trastuzumab-based adjuvant chemotherapy for early breast cancer. Intern Emerg Med. 2012 Oct;7(5):439-46. doi: 10.1007/s11739-012-0794-9. Epub 2012 Jun 20. PMID: 22714882.
15) Battisti NML, Andres MS, Lee KA, Ramalingam S, Nash T, Mappouridou S, Senthivel N, Asavisanu K, Obeid M, Tripodaki ES, Angelis V, Fleming E, Goode EF, John S, Rosen SD, Allen M, Stanway S, Lyon AR, Ring A. Incidence of cardiotoxicity and validation of the Heart Failure Association-International Cardio-Oncology Society risk stratification tool in patients treated with trastuzumab for HER2-positive early breast cancer. Breast Cancer Res Treat. 2021 Jul;188(1):149-163. doi: 10.1007/s10549-021-06192-w. Epub 2021 Apr 5. PMID: 33818652.
16) Bradshaw PT, Stevens J, Khankari N, Teitelbaum SL, Neugut AI, Gammon MD. Cardiovascular Disease Mortality Among Breast Cancer Survivors. Epidemiology. 2016 Jan;27(1):6-13. doi: 10.1097/EDE.0000000000000394. PMID: 26414938; PMCID: PMC4666721.
17) Castillo C, Camejo N, Etcheverria C, Ferradaz J, Ferreira A, Fontan A, Gabin AS, Herrera G, Artagaveytia N, Parma G, Delgado L. Trastuzumab-induced cardiotoxicity in early breast cancer over a 10-year period in Uruguay. Medicine (Baltimore). 2022 Jul 29;101(30):e29927. doi: 10.1097/MD.0000000000029927. PMID: 35905268; PMCID: PMC9333491.
18) Yuan C, Parekh H, Allegra C, George TJ, Starr JS. 5-FU induced cardiotoxicity: case series and review of the literature. Cardiooncology. 2019 Sep 6;5:13. doi: 10.1186/s40959-019-0048-3. PMID: 32154019; PMCID: PMC7048125.
19) Kanduri J, More LA, Godishala A, Asnani A. Fluoropyrimidine-Associated Cardiotoxicity. Cardiol Clin. 2019 Nov;37(4):399-405. doi: 10.1016/j.ccl.2019.07.004. Epub 2019 Aug 26. PMID: 31587781.
20) Polk A, Vaage-Nilsen M, Vistisen K, Nielsen DL. Cardiotoxicity in cancer patients treated with 5-fluorouracil or capecitabine: a systematic review of incidence, manifestations and

predisposing factors. Cancer Treat Rev. 2013 Dec;39(8):974-84. doi: 10.1016/j.ctrv.2013.03.005. Epub 2013 Apr 10. PMID: 23582737.
21) Deac AL, Burz CC, Bocsan IC, Buzoianu AD. Fluoropyrimidine-induced cardiotoxicity. World J Clin Oncol. 2020 Dec 24;11(12):1008-1017. doi: 10.5306/wjco.v11.i12.1008. PMID: 33437663; PMCID: PMC7769712.
22) Kosmas C, Kallistratos MS, Kopterides P, Syrios J, Skopelitis H, Mylonakis N, Karabelis A, Tsavaris N. Cardiotoxicity of fluoropyrimidines in different schedules of administration: a prospective study. J Cancer Res Clin Oncol. 2008 Jan;134(1):75-82. doi: 10.1007/s00432-007-0250-9. Epub 2007 Jul 17. PMID: 17636329.
23) Dobbin SJH, Cameron AC, Petrie MC, Jones RJ, Touyz RM, Lang NN. Toxicity of cancer therapy: what the cardiologist needs to know about angiogenesis inhibitors. Heart. 2018 Dec;104(24):1995-2002. doi: 10.1136/heartjnl-2018-313726. Epub 2018 Sep 18. PMID: 30228246; PMCID: PMC6268130.
24) Zamorano JL, Lancellotti P, Rodriguez Muñoz D, Aboyans V, Asteggiano R, Galderisi M, Habib G, Lenihan DJ, Lip GYH, Lyon AR, Lopez Fernandez T, Mohty D, Piepoli MF, Tamargo J, Torbicki A, Suter TM; ESC Scientific Document Group. 2016 ESC Position Paper on cancer treatments and cardiovascular toxicity developed under the auspices of the ESC Committee for Practice Guidelines: The Task Force for cancer treatments and cardiovascular toxicity of the European Society of Cardiology (ESC). Eur Heart J. 2016 Sep 21;37(36):2768-2801. doi: 10.1093/eurheartj/ehw211. Epub 2016 Aug 26. Erratum in: Eur Heart J. 2018 Mar 7;39(10):839. doi: 10.1093/eurheartj/ehw562. PMID: 27567406.
25) Touyz RM, Lang NN, Herrmann J, van den Meiracker AH, Danser AHJ. Recent Advances in Hypertension and Cardiovascular Toxicities With Vascular Endothelial Growth Factor Inhibition. Hypertension. 2017 Aug;70(2):220-226. doi: 10.1161/HYPERTENSIONAHA.117.08856. Epub 2017 Jun 19. PMID: 28630211; PMCID: PMC5509510.
26) Sonaglioni A, Albini A, Fossile E, Pessi MA, Nicolosi GL, Lombardo M, Anzà C, Ambrosio G. Speckle-Tracking Echocardiography for Cardioncological Evaluation in Bevacizumab-Treated Colorectal Cancer Patients. Cardiovasc Toxicol. 2020 Dec;20(6):581-592. doi: 10.1007/s12012-020-09583-5. PMID: 32519318.
27) Touyz RM, Lang NN, Herrmann J, van den Meiracker AH, Danser AHJ. Recent Advances in Hypertension and Cardiovascular Toxicities With Vascular Endothelial Growth Factor Inhibition. Hypertension. 2017 Aug;70(2):220-226. doi: 10.1161/HYPERTENSIONAHA.117.08856. Epub 2017 Jun 19. PMID: 28630211; PMCID: PMC5509510.
28) Postow MA, Sidlow R, Hellmann MD. Immune-Related Adverse Events Associated with Immune Checkpoint Blockade. N Engl J Med. 2018 Jan 11;378(2):158-168. doi: 10.1056/NEJMra1703481. PMID: 29320654.
29) Lyon AR, Yousaf N, Battisti NML, Moslehi J, Larkin J. Immune checkpoint inhibitors and cardiovascular toxicity. Lancet Oncol. 2018 Sep;19(9):e447-e458. doi: 10.1016/S1470-2045(18)30457-1. PMID: 30191849.
30) Dolladille C, Akroun J, Morice PM, Dompmartin A, Ezine E, Sassier M, Da-Silva A, Plane AF, Legallois D, L'Orphelin JM, Alexandre J. Cardiovascular immunotoxicities associated with immune checkpoint inhibitors: a safety meta-analysis. Eur Heart J. 2021 Dec 21;42(48):4964-4977. doi: 10.1093/eurheartj/ehab618. PMID: 34529770.

31) Johnson DB, Balko JM, Compton ML, Chalkias S, Gorham J, Xu Y, Hicks M, Puzanov I, Alexander MR, Bloomer TL, Becker JR, Slosky DA, Phillips EJ, Pilkinton MA, Craig-Owens L, Kola N, Plautz G, Reshef DS, Deutsch JS, Deering RP, Olenchock BA, Lichtman AH, Roden DM, Seidman CE, Koralnik IJ, Seidman JG, Hoffman RD, Taube JM, Diaz LA Jr, Anders RA, Sosman JA, Moslehi JJ. Fulminant Myocarditis with Combination Immune Checkpoint Blockade. N Engl J Med. 2016 Nov 3;375(18):1749-1755. doi: 10.1056/NEJMoa1609214. PMID: 27806233; PMCID: PMC5247797.
32) Mahmood SS, Fradley MG, Cohen JV, Nohria A, Reynolds KL, Heinzerling LM, Sullivan RJ, Damrongwatanasuk R, Chen CL, Gupta D, Kirchberger MC, Awadalla M, Hassan MZO, Moslehi JJ, Shah SP, Ganatra S, Thavendiranathan P, Lawrence DP, Groarke JD, Neilan TG. Myocarditis in Patients Treated With Immune Checkpoint Inhibitors. J Am Coll Cardiol. 2018 Apr 24;71(16):1755-1764. doi: 10.1016/j.jacc.2018.02.037. Epub 2018 Mar 19. PMID: 29567210; PMCID: PMC6196725.
33) Nielsen DL, Juhl CB, Nielsen OH, Chen IM, Herrmann J. Immune Checkpoint Inhibitor-Induced Cardiotoxicity: A Systematic Review and Meta-Analysis. JAMA Oncol. 2024 Oct 1;10(10):1390-1399. doi: 10.1001/jamaoncol.2024.3065. PMID: 39172480; PMCID: PMC11342217.
34) Gürdoğan M, Yalta K. Myocarditis associated with immune checkpoint inhibitors: Practical considerations in diagnosis and management. Anatol J Cardiol. 2020 Aug;24(2):68-75. doi: 10.14744/AnatolJCardiol.2020.79584. PMID: 32749254; PMCID: PMC7460679.
35) Salido Iniesta M, López López L, Carreras Costa F, Sionis A. A different type of acute myocarditis: a case report of acute autoimmune myocarditis mediated by anti-PD-1 T lymphocyte receptor (pembrolizumab). Eur Heart J Case Rep. 2020 Oct 4;4(5):1-6. doi: 10.1093/ehjcr/ytaa214. PMID: 33204987; PMCID: PMC7649507.
36) Palaskas N, Lopez-Mattei J, Durand JB, Iliescu C, Deswal A. Immune Checkpoint Inhibitor Myocarditis: Pathophysiological Characteristics, Diagnosis, and Treatment. J Am Heart Assoc. 2020 Jan 21;9(2):e013757. doi: 10.1161/JAHA.119.013757. Epub 2020 Jan 21. PMID: 31960755; PMCID: PMC7033840.
37) Mylvaganam R, Avery R, Goldberg I, Makowski C, Kalhan R, Villaflor V, Cuttica MJ. Adverse effects of immune checkpoint inhibitor therapies on right ventricular function and pulmonary arterial dilatation. Pulm Circ. 2021 Feb 9;11(1):2045894021992236. doi: 10.1177/2045894021992236. PMID: 33623668; PMCID: PMC7878999.
38) Pohl J, Totzeck M, Mincu RI, Margraf SM, Scheipers L, Michel L, Mahabadi AA, Zimmer L, Rassaf T, Hendgen-Cotta UB. Right ventricular and atrial strain in patients with advanced melanoma undergoing immune checkpoint inhibitor therapy. ESC Heart Fail. 2022 Oct;9(5):3533-3542. doi: 10.1002/ehf2.14094. Epub 2022 Jul 27. PMID: 35894541; PMCID: PMC9715888.
39) Liu J, Cao Y, Zhu K, Yao S, Yuan M, Kong X, Liu X, Li Y, Cui Y, Han X, Zhou X, Meng R, Shi H. Early evaluation of subclinical cardiotoxicity in patients with lung cancer receiving immune checkpoint inhibitors by cardiovascular magnetic resonance: a prospective observational study. Quant Imaging Med Surg. 2022 Oct;12(10):4771-4785. doi: 10.21037/qims-22-41. PMID: 36185042; PMCID: PMC9511417.
40) Xu A, Yuan M, Zhan X, Zhao G, Mu G, Wang T, Hu H, Fu H. Early detection of immune checkpoint inhibitor-related subclinical cardiotoxicity: A pilot study by using speckle tracking imaging and three-dimensional echocardiography. Front Cardiovasc Med. 2022

Dec 21;9:1087287. doi: 10.3389/fcvm.2022.1087287. PMID: 36620612; PMCID: PMC9812579.

41) Rubio-Infante N, Ramírez-Flores YA, Castillo EC, Lozano O, García-Rivas G, Torre-Amione G. Cardiotoxicity associated with immune checkpoint inhibitor therapy: a meta-analysis. Eur J Heart Fail. 2021 Oct;23(10):1739-1747. doi: 10.1002/ejhf.2289. Epub 2021 Jul 29. PMID: 34196077.
42) Salem JE, Manouchehri A, Moey M, Lebrun-Vignes B, Bastarache L, Pariente A, Gobert A, Spano JP, Balko JM, Bonaca MP, Roden DM, Johnson DB, Moslehi JJ. Cardiovascular toxicities associated with immune checkpoint inhibitors: an observational, retrospective, pharmacovigilance study. Lancet Oncol. 2018 Dec;19(12):1579-1589. doi: 10.1016/S1470-2045(18)30608-9. Epub 2018 Nov 12. PMID: 30442497; PMCID: PMC6287923.
43) Brumberger ZL, Branch ME, Klein MW, Seals A, Shapiro MD, Vasu S. Cardiotoxicity risk factors with immune checkpoint inhibitors. Cardiooncology. 2022 Mar 11;8(1):3. doi: 10.1186/s40959-022-00130-5. PMID: 35277208; PMCID: PMC8915459.
44) Chennamadhavuni A, Abushahin L, Jin N, Presley CJ, Manne A. Risk Factors and Biomarkers for Immune-Related Adverse Events: A Practical Guide to Identifying High-Risk Patients and Rechallenging Immune Checkpoint Inhibitors. Front Immunol. 2022 Apr 26;13:779691. doi: 10.3389/fimmu.2022.779691. PMID: 35558065; PMCID: PMC9086893.
45) Morelli MB, Bongiovanni C, Da Pra S, Miano C, Sacchi F, Lauriola M, D'Uva G. Cardiotoxicity of Anticancer Drugs: Molecular Mechanisms and Strategies for Cardioprotection. Front Cardiovasc Med. 2022 Apr 15;9:847012. doi: 10.3389/fcvm.2022.847012. PMID: 35497981; PMCID: PMC9051244.
46) Levey AS, Stevens LA, Schmid CH, Zhang YL, Castro AF 3rd, Feldman HI, Kusek JW, Eggers P, Van Lente F, Greene T, Coresh J; CKD-EPI (Chronic Kidney Disease Epidemiology Collaboration). A new equation to estimate glomerular filtration rate. Ann Intern Med. 2009 May 5;150(9):604-12. doi: 10.7326/0003-4819-150-9-200905050-00006. Erratum in: Ann Intern Med. 2011 Sep 20;155(6):408. PMID: 19414839; PMCID: PMC2763564.
47) Lang RM, Badano LP, Mor-Avi V, Afilalo J, Armstrong A, Ernande L, Flachskampf FA, Foster E, Goldstein SA, Kuznetsova T, Lancellotti P, Muraru D, Picard MH, Rietzschel ER, Rudski L, Spencer KT, Tsang W, Voigt JU. Recommendations for cardiac chamber quantification by echocardiography in adults: an update from the American Society of Echocardiography and the European Association of Cardiovascular Imaging. J Am Soc Echocardiogr. 2015 Jan;28(1):1-39.e14. doi: 10.1016/j.echo.2014.10.003. PMID: 25559473.
48) Nagueh SF, Smiseth OA, Appleton CP, Byrd BF 3rd, Dokainish H, Edvardsen T, Flachskampf FA, Gillebert TC, Klein AL, Lancellotti P, Marino P, Oh JK, Popescu BA, Waggoner AD. Recommendations for the Evaluation of Left Ventricular Diastolic Function by Echocardiography: An Update from the American Society of Echocardiography and the European Association of Cardiovascular Imaging. J Am Soc Echocardiogr. 2016 Apr;29(4):277-314. doi: 10.1016/j.echo.2016.01.011. PMID: 27037982.
49) Humbert M, Kovacs G, Hoeper MM, Badagliacca R, Berger RMF, Brida M, Carlsen J, Coats AJS, Escribano-Subias P, Ferrari P, Ferreira DS, Ghofrani HA, Giannakoulas G,

Kiely DG, Mayer E, Meszaros G, Nagavci B, Olsson KM, Pepke-Zaba J, Quint JK, Rådegran G, Simonneau G, Sitbon O, Tonia T, Toshner M, Vachiery JL, Vonk Noordegraaf A, Delcroix M, Rosenkranz S; ESC/ERS Scientific Document Group. 2022 ESC/ERS Guidelines for the diagnosis and treatment of pulmonary hypertension. Eur Heart J. 2022 Oct 11;43(38):3618-3731. doi: 10.1093/eurheartj/ehac237. Erratum in: Eur Heart J. 2023 Apr 17;44(15):1312. doi: 10.1093/eurheartj/ehad005. PMID: 36017548.

50) Nishimura RA, Otto CM, Bonow RO, Carabello BA, Erwin JP 3rd, Fleisher LA, Jneid H, Mack MJ, McLeod CJ, O'Gara PT, Rigolin VH, Sundt TM 3rd, Thompson A. 2017 AHA/ACC Focused Update of the 2014 AHA/ACC Guideline for the Management of Patients With Valvular Heart Disease: A Report of the American College of Cardiology/American Heart Association Task Force on Clinical Practice Guidelines. Circulation. 2017 Jun 20;135(25):e1159-e1195. doi: 10.1161/CIR.0000000000000503. Epub 2017 Mar 15. PMID: 28298458.
51) Voigt JU, Pedrizzetti G, Lysyansky P, Marwick TH, Houle H, Baumann R, Pedri S, Ito Y, Abe Y, Metz S, Song JH, Hamilton J, Sengupta PP, Kolias TJ, d'Hooge J, Aurigemma GP, Thomas JD, Badano LP. Definitions for a common standard for 2D speckle tracking echocardiography: consensus document of the EACVI/ASE/Industry Task Force to standardize deformation imaging. Eur Heart J Cardiovasc Imaging. 2015 Jan;16(1):1-11. doi: 10.1093/ehjci/jeu184. Epub 2014 Dec 18. PMID: 25525063.
52) Badano LP, Muraru D, Parati G, Haugaa K, Voigt JU. How to do right ventricular strain. Eur Heart J Cardiovasc Imaging. 2020 Aug 1;21(8):825-827. doi: 10.1093/ehjci/jeaa126. PMID: 32504092.
53) Voigt JU, Mălăescu GG, Haugaa K, Badano L. How to do LA strain. Eur Heart J Cardiovasc Imaging. 2020 Jul 1;21(7):715-717. doi: 10.1093/ehjci/jeaa091. PMID: 32548623.
54) Galderisi M, Cosyns B, Edvardsen T, Cardim N, Delgado V, Di Salvo G, Donal E, Sade LE, Ernande L, Garbi M, Grapsa J, Hagendorff A, Kamp O, Magne J, Santoro C, Stefanidis A, Lancellotti P, Popescu B, Habib G; 2016–2018 EACVI Scientific Documents Committee; 2016–2018 EACVI Scientific Documents Committee. Standardization of adult transthoracic echocardiography reporting in agreement with recent chamber quantification, diastolic function, and heart valve disease recommendations: an expert consensus document of the European Association of Cardiovascular Imaging. Eur Heart J Cardiovasc Imaging. 2017 Dec 1;18(12):1301-1310. doi: 10.1093/ehjci/jex244. PMID: 29045589.
55) Yingchoncharoen T, Agarwal S, Popović ZB, Marwick TH. Normal ranges of left ventricular strain: a meta-analysis. J Am Soc Echocardiogr. 2013 Feb;26(2):185-91. doi: 10.1016/j.echo.2012.10.008. Epub 2012 Dec 3. PMID: 23218891.
56) Muraru D, Onciul S, Peluso D, Soriani N, Cucchini U, Aruta P, Romeo G, Cavalli G, Iliceto S, Badano LP. Sex- and Method-Specific Reference Values for Right Ventricular Strain by 2-Dimensional Speckle-Tracking Echocardiography. Circ Cardiovasc Imaging. 2016 Feb;9(2):e003866. doi: 10.1161/CIRCIMAGING.115.003866. PMID: 26860970.
57) Pathan F, D'Elia N, Nolan MT, Marwick TH, Negishi K. Normal Ranges of Left Atrial Strain by Speckle-Tracking Echocardiography: A Systematic Review and Meta-Analysis. J Am Soc Echocardiogr. 2017 Jan;30(1):59-70.e8. doi: 10.1016/j.echo.2016.09.007. Epub 2016 Oct 27. PMID: 28341032.
58) Krittanawong C, Maitra NS, Hassan Virk HU, Farrell A, Hamzeh I, Arya B, Pressman GS, Wang Z, Marwick TH. Normal Ranges of Right Atrial Strain: A Systematic Review and

Meta-Analysis. JACC Cardiovasc Imaging. 2023 Mar;16(3):282-294. doi: 10.1016/j.jcmg.2022.06.022. Epub 2022 Sep 14. PMID: 36648033.

59) Huebner M, Vach W, le Cessie S. A systematic approach to initial data analysis is good research practice. J Thorac Cardiovasc Surg. 2016 Jan;151(1):25-7. doi: 10.1016/j.jtcvs.2015.09.085. Epub 2015 Sep 28. PMID: 26602896.

60) Koo TK, Li MY. A Guideline of Selecting and Reporting Intraclass Correlation Coefficients for Reliability Research. J Chiropr Med. 2016 Jun;15(2):155-63. doi: 10.1016/j.jcm.2016.02.012. Epub 2016 Mar 31. Erratum in: J Chiropr Med. 2017 Dec;16(4):346. doi: 10.1016/j.jcm.2017.10.001. PMID: 27330520; PMCID: PMC4913118.
