# Supplementary material for: Comorbidity Burden and Cumulative Anti-HER2 Exposure Associated with Cardiac Dysfunction in Breast Cancer: A Real-World Cardio-Oncology Cohort
Source: J Clin Med. 2026 Oct 5;15(19):7700. doi: 10.3390/jcm15197700 (PMC13665819; doi:10.3390/jcm15197700)
Supplement: Supplementary file 1 [file jcm-15-07700-s001.zip › Supplementary Material File S2.pdf]

09/05/2026

In riferimento allo studio **CARTOX-PREDICT** , avente per titolo:

Cardiotossicità nei pazienti oncologici trattati con terapie antitumorali moderne: studio retro-prospettico monocentrico.

cui è condizionato il Parere Etico espresso nella seduta del 18.11.2025 si rivaluta la seguente documentazione pervenuta in data 30.04.2026

Lettera di trasmissione Lettera-2nda-Risposta-CET_CARTOX-PREDICT.pdf Lettera di tramissione riscontro alla comunicazione CE - chiarimenti - del 22.1.2026
Protocollo 01_Protocollo-CARTOX-PREDICT_V1.1_07.10.2025_CL.pdf 01_Protocollo-CARTOX-PREDICT_V1.1_07.10.2025_TC.pdf Protocollo CARTOX-PREDICT_V1.1_07.10.2025 TC e clean
Informativa e consenso base 03_Foglio-Informativo_-CARTOX-PREDICT_V1.1_07.10.2025_CL.pdf 03_Foglio-Informativo_-CARTOX-PREDICT_V1.1_07.10.2025_TC.pdf Foglio Informativo_ CARTOX-PREDICT_V1.1_07.10.2025 TC e clean
Altro CVRISK-IT_LETTERA-RICHIESTA-EMENDAMENTO_n.4_PSD.pdf
Dichiarazione_sostitutiva_di_modulo_consenso_CARTOX-PREDICT_SONAGLIONI_firmato-PI-21.04.2026.pdf Dichiarazione sostitutiva modulo di consenso - Nuova versione 21.4.2026

Presa visione della documentazione. che recepisce le richieste di modifica avanzate del nostro CET Lombardia 5, viene sciolta la riserva.

Il Segretario
Dott. Michele Tedeschi

Michele
Tedeschi
08.05.2026
23:44:12
GMT+01:00
